# Binding of Glutamic Acid to Silver and Gold Nanoparticles Investigated by Surface-Enhanced Raman Spectroscopy

**DOI:** 10.3390/bios14110522

**Published:** 2024-10-25

**Authors:** Vlasta Mohaček-Grošev, Marko Škrabić, Hrvoje Gebavi, Vesna Blažek Bregović, Ivan Marić, Vincenzo Amendola, Jože Grdadolnik

**Affiliations:** 1 Center of Excellence for Advanced Materials and Sensing Devices, Research Unit New Functional Materials, Ruđer Bošković Institute, Bijenička Cesta 54, 10000 Zagreb, Croatia; hrvoje.gebavi@irb.hr; 2 Department of Physics and Biophysics, School of Medicine, University of Zagreb, Šalata bb, 10000 Zagreb, Croatia; marko.skrabic@mef.hr; 3 Laboratory for Optics and Thin Films, Division of Materials Physics, Ruđer Bošković Institute, Bijenička Cesta 54, 10000 Zagreb, Croatia; vblazek@irb.hr; 4 Radiation Chemistry and Dosimetry Laboratory, Ruđer Bošković Institute, Bijenička Cesta 54, 10000 Zagreb, Croatia; imaric@irb.hr; 5 Department of Chemical Sciences, University of Padova, I-35131 Padova, Italy; vincenzo.amendola@unipd.it; 6 National Institute of Chemistry, Hajdrihova 19, 1000 Ljubljana, Slovenia

**Keywords:** glutamate, glutamic acid, surface-enhanced Raman spectroscopy, AuNP, AgNP, CRYSTAL09, normal modes

## Abstract

Glutamate is the most important excitatory neurotransmitter, which is relevant for the study of several diseases such as amyotrophic lateral sclerosis and Alzheimer. It is the form L-glutamic acid (Glu) takes at physiologically relevant pHs. The surface-enhanced Raman spectra of Glu obtained at pH values ranging from 3.3 to 12 are collected in the presence of silver and gold colloids and on solid substrates. The observed bands are compared with the positions of calculated normal modes for free neutral glutamic acid, glutamic acid monohydrate, glutamic acid bound to gold and silver atoms, and sodium glutamate. Although gold atoms prefer to bind to the NH_2_ group as compared to carbonyl groups, silver atoms prefer binding to hydroxyl groups more than binding to the amino group. SERS spectra of glutamic acid solutions with a pH value of 12, in which both carboxylic groups are deprotonated, indicate a complexation of the glutamic acid dianion with the sodium cation, which was introduced into the solution to adjust the pH value. Further research towards an optimal substrate is needed.

## 1. Introduction

The dianion of glutamic acid—glutamate—serves as a neurotransmitter in humans. It is released from synaptic vesicles and taken up by neurons or astrocytes to terminate the signal [1]. Glutamate is the most important excitatory neurotransmitter in the central nervous system of mammals and the most abundant free amino acid in the brain [2]. In emergency situations, cerebrospinal fluid samples are taken from patients to determine glutamine levels in order to diagnose uncontrollable epileptic seizures [3]. The detection and measurement of extracellular glutamate levels is of great practical importance, as excess glutamate can lead to excitotoxicity and contribute to conditions such as amyotrophic lateral sclerosis and Alzheimer’s disease [4]. In addition, glutamate concentration is an indicator of central nervous system injury [5] and is sensitive to it.

Currently, the most accurate laboratory methods used for monitoring glutamate levels are high-performance liquid chromatography (HPLC) and gas chromatography-mass spectrometry (GCMS), while enzymatic electrochemical sensors can detect and/or quantify glutamate in some biofluids [4]. Nonenzymatic electrochemical sensors based on metal nanoparticles have not yet reached the required detection limit (glutamate concentrations in plasma are between 1 and 100 µM, but variations from 1 µM to 10 µM in cerebrospinal fluid are important [1]. Optical sensing methods such as surface-enhanced Raman spectroscopy (SERS) utilize metal nanoparticles, either in colloids or on substrates, to facilitate the creation of hotspots for analyte binding [6,7]. Detection of pharmaceutical pollutants [8,9], fluoride anions [10], or antimicrobial agents of the hydrazone type [11] were reported, together with herbicides such as di- or trichlorophenoxyacetic acid [12]. Designers of SERS substrates aim at quantifying the nanoparticle concentration [13] or at developing 2D materials such as SnS_2_, MoS_2_, or WSe_2_ [14]. Studies where SERS was used for the detection of artificial dyes to screen additives in food [15] or pigments in artwork preservation were published [16]. Model molecules are often chosen for studying the SERS effect in detail, for example, 4-aminothiophenol [17] or pyridine [18]. Theoretical modeling approximates metal nanoparticles or substrates by Ag_n_ or Au_n_ clusters or slabs [9,10,12,16,18,19]. A review by Jensen et al. dedicated to modeling the SERS mechanism has recently been published [20].

Raman spectroscopists have been interested in amino acids both in their crystalline and liquid states for quite some time [21,22,23,24,25,26,27]. The detection limit of amino acids in aqueous solution at acidic pH by standard Raman spectroscopy was set at 7 mM by Numata et al. [28]. One of the first reports on vibrational analysis of L-glutamic acid (Glu) was that of Shurvell and Bergin on Raman spectra of saturated aqueous solutions at pH 0.5 to 12.5, including spectra of polycrystalline L-Glu and monosodium glutamate [29]. Dhamelincourt and Ramírez obtained polarized micro-Raman and FTIR spectra of solid Glu [30]. López Navarrete et al. provided infrared and Raman spectra for deuterated L-Glu and ^15^N-L-Glu [31], while Ramírez and López Navarrete performed normal coordinate calculation for the neutral form of glutamic acid [32]. The infrared spectra of the neutral form of Glu isolated in an argon matrix were compared with the scaled 4–31 G frequencies calculated ab initio in a later publication by López Navarrete et al. [33]. More recently, Yuan et al. studied the spectral and dissociation processes of Glu in an external electric field [34], while Voges et al. investigated the solubility of L-Glu in aqueous solutions in dependence on pH [35]. Williams et al. analyzed peptide fragments containing L-glutamic acid both by Raman spectroscopy and computationally using DFT methods [36]. It has been found that polyglutamic acid in the form of octamers can bind to metallic nanoparticles [37,38,39,40,41]. Sodium polyglutamate polymer can reversibly change its conformation in water [42].

Of greater interest to us is the ability of a single Glu molecule to bind to a metal nanoparticle, whether in a colloid or on a solid substrate. In their pioneering study on silver colloids, Suh and Moskovits interpreted spectra of glycine and alanine solutions with a pH of 6.92 as originating from binding of amino acids to silver nanoparticles (AgNPs) with both amino- and carboxy termini [43]. Chumanov et al. [44] presented the SERS spectrum of glutamic acid at neutral pH for the first time and observed the strongest band at 1367 cm^−^^1^, which they assigned to the symmetric stretching of the COO^-^ group. Further bands occurred at 620, 830, 952, 1048, and 1230 cm^−^^1^.

Stewart and Fredericks were able to record SERS spectra of 19 amino acids, including glutamic acid, using an electrochemically prepared silver surface [45], with the proposed binding via the –COO^−^ group. Xiaoming Dou et al. investigated the effects of pH on the binding of glycine to gold [46] and silver nanoparticles [47]. They reported rapid glycine coagulation at a pH of 3.9 in silver colloids. However, the silver colloid they prepared using the Creighton method contained positively charged metal particles, while other groups reported negatively charged particles [48,49]. The Creighton method was also used by O’Neal et al. to study micromolar glutamate concentrations detectable with SERS [5]. The band at 830 cm^−^^1^ was filtered out as the one that could help distinguish glutamic from aspartic acid [5]. SERS spectra of basic solutions of L-glycine, L-proline, L-cysteine, L-phenylalanine, and their dipeptides were obtained by Podstawka et al. [50]. Again, the -COO^-^ symmetric stretching band at 1388 cm^−^^1^ was the strongest band in the SERS spectrum of glycine at pH 9.8, and the middle band at 1036 cm^−^^1^ could be explained as the C-N stretching of the –NH_2_ group bound to the silver particle surface [50]. Sengupta et al. [51] applied SERS to characterize bioaerosol and chose D-glutamic acid, D-alanine, and L-lysine as three amino acids present in bacterial walls. All three amino acids mentioned yielded very similar spectra, with bands of glutamic acid being most intense at 1640, 1401, and 1379 cm^−^^1^ [51].

Seventeen amino acids, including glutamic acid, at a concentration of 6.8 mM were analyzed by Guicheteau et al. using both normal Raman and SERS for bacterial identification [52]. The authors used solutions mixed with silver colloids, and the SERS spectra were recorded after the droplets dried on aluminum slides [52]. Sawai and coworkers [53] applied an electric field of 20 V/cm to the silver film on a glass substrate immersed in a solution of 1 mM glutamic acid. They provided SERS spectra for the concentration range from 1 nM to 1 mM and showed the time-dependent spectral changes for 1 μM concentration. Daizy Philip [54] opted for citrate-reduced gold nanoparticles with a size of approximately 35 nm and tested them with glutamic acid. The most prominent bands she observed were at 1365, 1239, and 1008 cm^−^^1^, corresponding to –COO^-^ sym. stretching, δ(CH2) wagging, and C–N stretching vibrations, respectively. More recently, Lee et al. used frequency modulation of the exciting laser beam and detected SERS signals in order to better distinguish SERS spectra of attomolar concentrations of glutamate and other neurotransmitters from the background noise [55].

Since published results on SERS spectra of glutamic acid demonstrated a large diversity of observed Raman bands, we undertook an investigation of the binding of glutamic acid to the surface of silver and gold nanoparticles in order to gain a better understanding of the relation of the spectroscopic signals with the chemical state of the analyte. Also, since most previous studies used colloids, we tested some of the commercial substrates available in order to check the similarity of observed SERS spectra with that from the literature.

This study was supported with the ab initio calculation of normal modes of free glutamic acid, glutamic acid monohydrate, glutamic acid bound to gold and silver atoms, and sodium glutamate and vibrations of glutamic acid in the crystalline state where it takes zwitterionic form. The purpose of calculations was to estimate the extent of shifting of vibrational modes with respect to those of free molecules and to obtain a more confident assignment of the observed SERS bands. Previous calculations of vibrational modes were performed for a free molecule only.

## 2. Materials and Methods

The powder of polycrystalline L-glutamic acid with purity >99% was purchased from Kemika d.d., Zagreb, Croatia, and used without further purification. The polymorph was identified as β-glutamic acid using Raman measurements [56]. The first stock solution with a concentration of 10 mM was prepared by weighing 73.5 mg of powder and mixing it with 50 mL of extrapure water demineralized to a conductivity of 0.055 μS/cm using the SG RO 6 Sp ultrapure water system. The pH of the solution was 3.5. The second stock solution of 10 mM was prepared using MQ water by the Stakpure OmniaLab DS 60 instrument, and its pH was 3.3. From these solutions, all other solutions were prepared by proportional dilution.

The pH of the solutions was determined using an Edge Blue pH meter from Hanna Instruments. Solutions with a pH of 3.3 and 3.5 correspond to water solutions of Glu prepared in two different series of experiments. Solutions having pH values of 7, 10, or 12 were prepared by adding appropriate amounts of NaOH.

Silver and gold colloids were prepared by laser ablation in liquid to avoid the presence of contaminants such as organic stabilizers on the surface of the NPs. The NPs were obtained by laser ablation synthesis using 1064 nm laser pulses (6 ns, 50 Hz) from a Q-switched laser focused with a lens of f = 100 mm to a fluence of 5 J/cm^2^ on a 99.99% pure metal plate of Au or Ag immersed in a 10^−^^4^ M NaCl solution in double-distilled water [57,58].

Gold and silver colloids were prepared for transmission electron microscopy according to a previously described procedure [59]. The average size of silver particles was 26 ± 6 nm and of gold particles was 15.5 ± 3.9 nm (Figures S1 and S2 in the Supporting Information).

**Figure 1:**
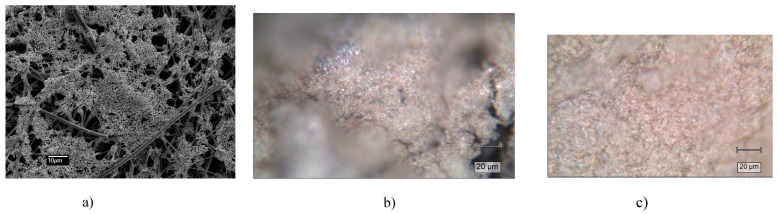
(**a**) Scanning electron microscopic image of the OceanInsight RAM-SERS Ag substrate at 1000× magnification. The bar is 10 µm. (**b**) Optical image of the OceanInsight RAM-SERS Ag substrate, where the bar is 20 µm. (**c**) Optical image of the SersitiveAgAu substrate, where the bar is 20 µm.

The morphology of the purchased Ocean Insight 532 nm substrates was examined using the Jeol JSM 7000F scanning electron microscope (SEM) at 10 kV and 1000× or 15,000× magnification (Figure 1).

Four samples were prepared for absorbance in the UV–VIS region by taking 200 µL of each sample (pure AuNPs 0.135 mg/mL, pure AgNPs 0.016 mg/mL, 1 mM pure water solution of glutamic acid, and 10 mM water solution of Glu with a pH of 12) and diluted with 2 mL of MQ water in a quartz cuvette with an optical path length of 1 cm, which was then placed in the scattering chamber of the Perkin Elmer Lambda 25 UV–VIS spectrometer. The spectra were recorded at the interval of 190–1000 nm. Mixtures of Glu and colloids were prepared by mixing 200 µL of Glu and 200 µL of Ag or Au colloid with 2 mL of MQ water.

Dynamic light scattering (DLS) experiments with 1 mM and 10 mM solutions were performed on samples at pH 3.5 and 12. The average hydrodynamic diameters of pure AuNPs and Glu-AuNP particles in 1:1 *v*/*v* water mixtures were measured using Malvern Panalytical’s Zetasizer Ultra instrument equipped with a 632.8 nm He-Ne laser and utilizing multiangle dynamic light scattering (MADLS) technology. MADLS performs the analysis at three different scattering angles (174.70, 90.00, and 12.780 degrees) and summarizes the data into a single integrated measurement. The measurements were performed in DTS0012 standard 10 mm diameter plastic cells. The hydrodynamic diameters were calculated based on intensity distributions. The results are given as averages of 3 measurements. Zeta potential measurements were performed by electrophoretic light scattering in folded capillary cells DTS1080. The values of the zeta potentials are given as the mean of three measurements.

Two types of commercial substrates were used, one with silver—OceanInsight RAM-SERS-Ag type and the other with Ag and Au (SersitiveAgAu substrate) (Figure 1). They were chosen because spectra of bare substrates either had no bands between 1500 cm^−^^1^ and 2800 cm^−^^1^ (OceanInsight) or the bands were very weak (Sersitive). A droplet of 5 μL of solution was left to dry on the substrate before measurement was undertaken. In every spectrum of the bare substrate, a strong band at 236 cm^−^^1^ was observed, indicating binding of metal nanoparticles to the layer below.

Fourier-transform infrared spectra of polycrystalline Glu mixed with KBr pressed into pellets were recorded with a Spectrum GX at the interval of 370–4000 cm^−^^1^ with a resolution of 4 cm^−^^1^ and 20 repetitions, using corrections for the subtraction of the spectrum of the surrounding water vapor.

Two spectrometers were used for the Raman measurements. The first spectrometer used was a T64000 Horiba JobinYvon Raman spectrometer in triple subtractive mode with green excitation by a 532 nm laser under a wide-angle objective with 50× magnification. The second spectrometer used was a Renishaw InVia with a 20× or 50× objective and excitation lasers of 532 nm and 785 nm. The laser power was mostly 0.3 mW, and the accumulation time was 1 to 5 s with four to sixteen repetitions. The baseline of all spectra was subtracted using either LabSpec 5 or the Wire 5.5 program. The Raman spectrum of a 1 mM solution in a metal container was also recorded to check the sensitivity of the system. With four repetitions and an accumulation time of 4 s, no vibrational bands other than those of water were detected. When recording spectra of colloids, a droplet of a 1:1 *v*/*v* mixture of Glu solution and a colloid was put on a silicon substrate. Therefore, the 520.7 cm^−^^1^ silicon band is visible in the spectra of colloids.

### Computational Methods

The normal modes of free Glu, Glu monohydrate, sodium glutamate, Glu-Au, and Glu-Ag molecules were calculated by optimizing the geometry and then calculating the frequencies using the Gaussian 16 program suite with B3LYP functional [60]. At first, 6–31++G(d,p) basis set was used for Glu, Glu-H_2_O, and sodium glutamate. For Glu-Au and Glu-Ag, the lanl2dz basis set was chosen, and the geometry optimization for Glu and Glu-H_2_O was repeated with this basis set to compare the binding energies of Glu to water and metal atoms. A selected list of normal modes that are most illustrative of Glu binding, scaled by a factor of 0.968, is shown in Table 1. All frequencies were found to be positive (see Tables S1–S5 in the Supporting Information). The scaling factor was chosen from the ratio of the calculated wavenumber for the symmetric COO^−^ stretch of sodium glutamate (1437 cm^−^^1^) and the observed band at 1392 cm^−^^1^ in the SERS spectrum of Glu at pH 12.

**Figure 2:**
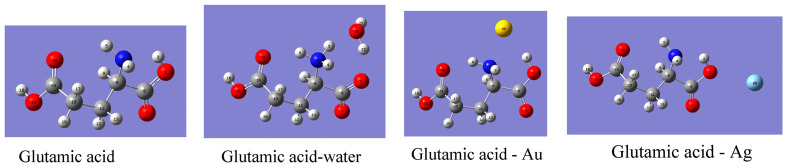
Optimized structures of glutamic acid, glutamic acid monohydrate, and glutamic acid bound to gold and silver atoms for which normal modes were calculated at the b3lyp/lanl2dz level of theory (see Tables S1–S4 in the Supporting Information).

The stable configurations of neutral glutamic acid, Glu-H_2_O, Glu-Au, and Glu-Ag are shown in Figure 2. All configurations, including sodium glutamate depicting atom notations, are available within Supplementary Information. There exists an intramolecular hydrogen bond O1-H3···N2 in neutral Glu that manifests itself in different values of O1-H3 stretching vibration compared to ν(O14-H18), which is predicted lower by 370 cm^−^^1^ (Table S1). Also, the value of ν(C6=O7) is predicted at 1691 cm^−^^1^, compared to 1633 cm^−^^1^ predicted for ν(C19=O10). The O=C-O bending vibrations are also expected at different wavenumbers for two carboxyl groups as follows: δ(O7=C6-O1) at 561 cm^−^^1^ and δ(O10=C19-O14) at 526 cm^−^^1^.

While searching for stable configurations of gold atom bound to Glu, two configurations were obtained as follows: the first with the Au atom closest to carbonyl O7, which gave E_opt_ = −686.995775 Ha and the energy of Au-Glu binding −0.00714 Ha = −0.194 eV, and the second one where the gold atom was closest to the NH_2_ group. The optimization energy of the second configuration was E_opt_ = −686.998604 Ha, and the binding energy of Au was equal to −0.997 Ha = −0.271 eV. For the second configuration, normal modes were calculated (Figure 2 and Table S3).

Considering the binding of the silver atom, again it was found that Ag can form a stable configuration either by approaching the NH_2_ group or bonding to the hydroxyl O1 atom. The optimization energy of Glu-Ag when Ag was closest to NH_2_ was −697.309048 Ha, and the energy of Ag-Glu binding was −0.0015 Ha = −0.041 eV. When the silver atom was closest to O1, the E_opt_ = −697.311979 Ha, and the Glu···Ag binding energy was −0.00444 Ha = −0.121 eV. For the more stable Glu-Ag configuration, we calculated normal modes (Figure 2 and Table S4). We can conclude that both gold and silver atoms can bind to amino and carboxyl groups; Au prefers to bind to the amino group, and Ag prefers to bind to the hydroxyl oxygen atom of the carboxyl group closer to NH_2_.

The remaining question is as follows: how does binding of a single water molecule affect glutamic acid? Again, a search for stable Glu···H_2_O configurations was conducted, and two stable configurations were found. In the first configuration, one water molecule forms a hydrogen bond to carbonyl O7, and the Eopt = −627.976346 Ha. The binding energy of water to Glu (here it remains in neutral form) was −0.01317 Ha = −0.358 eV. The optimization energy for the second, more stable configuration where water binds to the NH_3_^+^ group was E_opt_ = −627.980744 Ha, and the binding energy of water was −0.01757 Ha = −0.476 eV. For the second configuration, normal modes were calculated (see Figure 2, Table S2, and Supporting Information).

The partial optimization of the atomic positions of crystalline β-polymorphic glutamic acid with fixed cell parameters was initially carried out using density functional theory, which is implemented in the CRYSTAL09 program [61]. The correlation functional of Lee, Yang, and Parr [62] with generalized gradient approximation and the exchange functional of Becke [63], commonly known as the B3LYP functional, were used. The basis functions for oxygen, carbon, hydrogen, and nitrogen atoms were taken from the study by Gatti et al. [64]. The parameters of the unit cell were a = 5.1586 Å, b = 6.9477 Å, c = 17.2861 Å, and α = β = γ = 90°, with Z equal to 4 [65]. The positions of the atoms within the unit cell were optimized using the keyword OPTGEOM, with the old wave function mixed 70% with the new experimental function (keyword FMIXING). Each molecule has 19 atoms, resulting in 76 atoms in the unit cell and 225 optical phonons that are all active in Raman and 168 of them in infrared spectra. In Table 2, calculated vibrations in the interval 1350–1780 cm^−^^1^ are presented, while insight into every vibrational motion can be obtained by uploading the CRYSTAL09 output to an online website [66].

## 3. Results and Discussion

**Figure 3:**
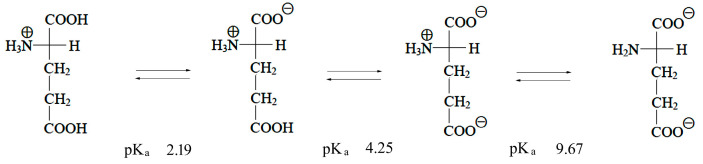
Scheme depicting transformations of L-glutamic acid at its characteristic pKa values.

A scheme depicting the protonation state of Glu at three different pH values is shown in Figure 3 [67].

**Figure 4:**
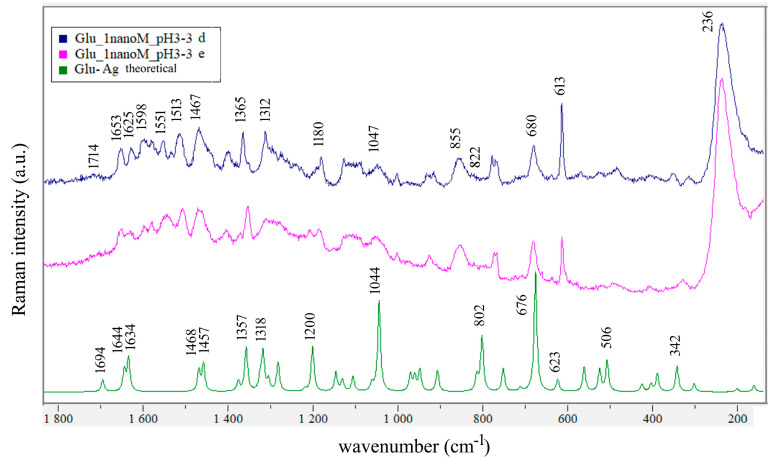
Comparison of the SERS spectra of 1 nM Glu at pH 3.3 on OceanInsight Ag substrate (**top** and **middle**) with the calculated scaled (0.968) theoretical SERS spectrum of Glu-Ag at the bottom. Laser excitation: 532 nm.

In Figure 4, two SERS spectra of 1 nM solution having pH 3.3 and the theoretical calculated SERS spectrum of Glu-Ag are compared. The spectra contain more bands in the 1500–1700 cm^−1^ interval than one would expect if there were only one type of binding site and one molecular species in solution. Specifically, for solution with pH 3.3, one expects one C=O stretching vibration, one antisymmetric stretching of CO_2_^-^, and three NH_3_^+^ bending vibrations per one type of binding site, and from Figure 4, one can immediately see that there are eight observed bands in that interval (see Table 3).

Our intention is to correlate the observed bands in the SERS spectra with the different protonation states of the carboxyl and amino groups. For that purpose, we compared calculated vibrational spectra of neutral Glu, polycrystalline Glu, Glu-H_2_O, Glu-Au, and Glu-Ag (see Table 1 and Table 2). The stable polymorph of Glu crystallizes in the space group *P2_1_2_1_2_1_* with four molecules per unit cell [65] and has the structure of zwitterion, displayed in Figure 3 as the structure between pKa 2.19 and 4.25 [67].

**Figure 5:**
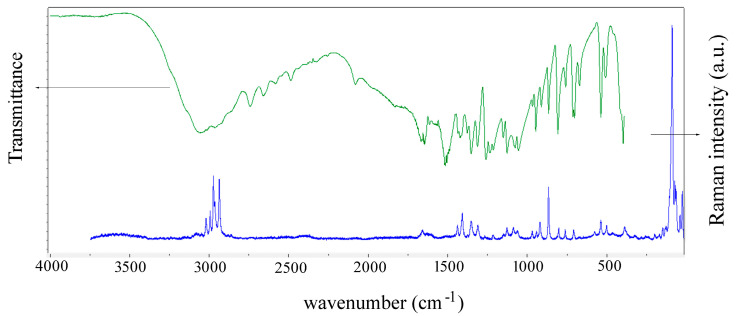
Infrared (370–4000 cm^−1^) and Raman (7–3700 cm^−1^) spectra of L-Glu in the polycrystalline form. Raman excitation: 532 nm.

Although the molecule is neutral, charged groups such as –NH_3_^+^ and –COO^−^ interact via Coulombic interactions, which promotes the stability of the crystal. Rotations coupled with translations along the three perpendicular crystal axes generate four symmetrically equivalent molecules in the unit cell of the crystal. All vibrational modes are active in the Raman spectrum (A, B_1_, B_2_, and B_3_), while B_1_, B_2_, and B_3_ are active in the infrared spectra of the polycrystalline sample. The experimental spectra are shown in Figure 5.

In Table 2, positions of calculated and observed bands for glutamic acid in the crystalline state and sodium glutamate (Na-Glu) in the 1350–1780 cm^−1^ interval are compared. Glu in the form of crystalline powder was determined to be in the form of β-glutamic acid polymorph [56,65]. The description of the calculated crystal modes in Table 2 shows that the ν(COO^−^) and ν(C=O) modes are coupled with the δ(NH_3_^+^) bending modes of all molecules in the unit cell. The ν(C=O) modes, although mixed in nature, are expected in the interval 1724–1735, while the asymmetric stretching ν_asym_(COO^−^) coupled to the δ(NH_3_^+^) bending is predicted in the intervals 1701–1707 cm^−1^ and 1626–1649 cm^−1^. The symmetric stretching ν_sym_(COO^−^) couples with δ(HCC) in the range of 1389–1398 cm^−1^. From the description of the calculated modes given in Table 2, one expects rather pure δ(NH_3_^+^) bending modes in the 1687–1692 cm^−1^ and 1570–1582 cm^−1^ intervals. The observed strong infrared bands for Glu powder at 1663 and 1645 cm^−1^ are attributed to the δ(NH_3_^+^) + ν_asym_(COO^−^) and δ(NH_3_^+^) modes, and the same assignment applies to the strong Raman bands at 1632 cm^−1^ and the strong infrared bands at 1615 cm^−1^. The nature of vibrations we obtained in Table 2 agrees with the sequence of normal modes obtained for zwitterionic Glu by Ramírez and López Navarrete [32].

Considering now normal coordinate analysis of free glutamic acid, we find it possesses an intramolecular hydrogen bond (Figure 2), which was not mentioned in the earlier ab initio study by López Navarrete et al. [33] who used a 4–31 G basis set in addition to semiempirical MNDO and AM1 methods. Based on those calculations, López Navarrete et al. assigned the 1781 and 1635 cm^−1^ bands observed in the FTIR spectrum of glutamic acid in the argon matrix as carbonyl stretching (which they calculated at 1778 and 1765 cm^−1^) and δ(NH_2_) bending vibration (theoretical value at 1634 cm^−1^). Also, they observed a band at 612 cm^−1^ and assigned it to δ(O=C–O) in plane bending (their calculated values for the two corresponding modes were 618 and 599 cm^−1^). Our results predict two carbonyl stretching modes, the ν(C6=O7) being 58 wavenumbers higher than ν(C19=O10) (Table 1). At the lower end of the spectrum, two δ(O=C–O) bending modes are predicted at 561 and 526 cm^−1^, while two δ(O=C-C) modes are expected at 504 and 422 cm^−1^. The 561 and 504 cm^−1^ modes are associated with the C6=O7 bond and the 526 and 422 cm^−1^ modes with the C19=O10 group. Our computations predict that binding of gold to amino group will reduce the ν(C6=O7) by 28 cm^−1^ and introduce mixing of δ(NH_2_) and ν(C19=O10) modes (Table 1). For Glu-Au, the bending mode δ(O7=C6–O1) is predicted at 555 cm^−1^, δ(O10=C19–O14) at 532 cm^−1^, δ(O7=C6–C8) at 476 cm^−1^, and δ(O10=C19C15) at 429 cm^−1^. The silver atom was found to prefer binding to hydroxyl oxygen O1, and the bending mode δ(O7=C6–O1) calculated at 676 cm^−1^ agrees with the observed one at 680 cm^−1^ (Figure 4 and Table 1). A δ(HNC) bending mode at 814 cm^−1^ and a ν(C–C) stretching mode at 802 cm^−1^ are predicted for Glu-Ag (Table S4).

**Figure 6:**
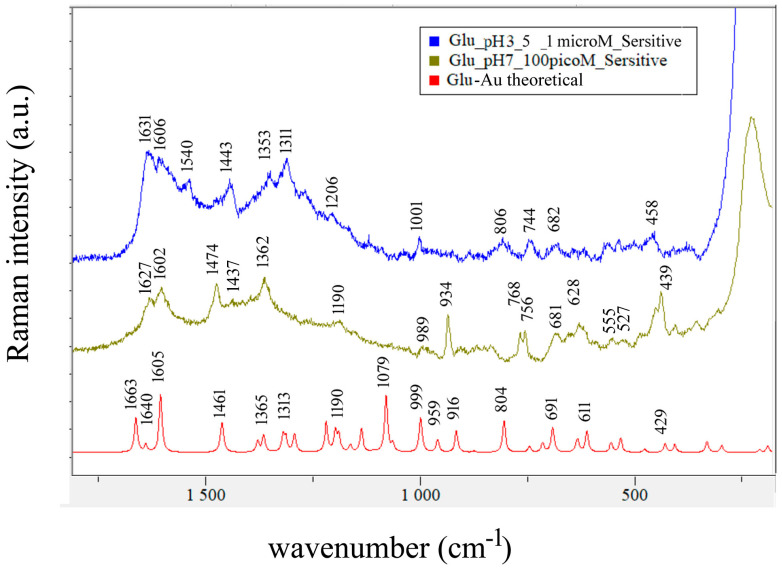
Comparison of the SERS spectrum of 1 μM Glu at pH 3.5 (**top**) and the SERS spectrum of 100 pM Glu at pH 7 (**middle**), both on SersitiveAgAu substrate, with excitation at 785 nm. Calculated scaled (0.968) theoretical SERS spectrum of Glu-Au is at the bottom.

**Figure 7:**
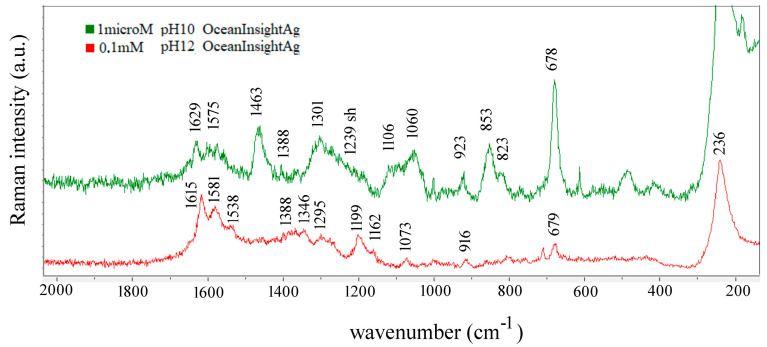
Comparison of the SERS spectrum of 1 μM Glu at pH 10 with the SERS spectrum of 0.1 mM Glu at pH 12. Both spectra were recorded with 532 nm on OceanInsight Ag substrate.

In Table 3, one can compare assignments of observed bands in SERS spectra of Glu at pH 3.3, 7, and 10, as displayed in Figure 4, Figure 6, and Figure 7.

The carbonyl stretching vibration appears at 1732 cm^−1^ in the Raman spectrum of the polycrystalline powder and at 1714 cm^−1^ in the SERS spectrum of Glu at pH 3.3 (Figure 4). For Glu at pH 7, one expects two antisymmetric stretching of CO_2_ and three NH_3_^+^ bending vibrations per one type of binding site, while strong bands at 1627, 1602, and 1562 cm^−1^ appear on top of a broad band centered roughly at 1600 cm^−1^. Shurvell and Bergin assigned the 1610 cm^−1^ band observed in the aqueous solution of Glu at pH 7 and the 1570 cm^−1^ band observed in the spectrum of solution having pH 12.5 to the asymmetric COO^−^ stretching vibration [29]. They are consistent with the weak band of the sodium acetate solution at pH 14 [68,69]. In contrast, if the calculated normal modes of sodium glutamate are considered, scaled values of δ(NH_2_) scissoring at 1621 cm^−1^ and ν_asym_(COO^−^) at 1539 cm^−1^ are found. The bands at 1615 and 1538 cm^−1^ observed in the SERS spectrum of 0.1 mM Glu at pH 12 we can assign to these modes (Figure 7 and Figure S3 in the Supplementary Information).

Differences in the observed spectra of Glu take place on changing pH and/or concentration. While calculated Raman spectra of Glu-Ag and Glu-Au served as a basis for the assignment presented in Table 3, the fact is that they were calculated for Glu in the neutral form, forming complexes with noble metal atoms, while SERS spectra were obtained at pH values at which at least one carboxyl group is deprotonated. Taking this into account, we assign a 1400 cm^−1^ medium and a strong 1365 cm^−1^ band observed in the 1 nanoM spectrum of Glu at pH 3.3, 1398, and 1365 cm^−1^ bands found in the spectrum of 0.1 nanoM spectrum of Glu at pH 7 (Figure 6) and 1388 cm^−1^ band observed in the spectrum of 1μM spectrum of Glu at pH 10 to the symmetric stretching of COO^−^ (Figure 7 and Table 3). Suh and Moskovits assigned the 1371 and 1361 cm^−1^ bands observed in the SERS spectrum of glycine to ν_sym_(COO^−^) [43], while Chumanov et al. assigned this mode to the 1367 cm^−1^ band in the SERS spectrum of Glu [44].

**Table 3:** Comparison of SERS spectra of glutamic acid at pH 3.3, 7, and 10 shown in Figure 4, Figure 6, and Figure 7. The assignment considers shifts of hydrated glutamic acid due to binding to Ag or Au.

1 nanoM pH 3.3 OceanInsight Ag 532 nm	Assignment	0.1 nanoM pH 7 Sersitive AgAu 785 nm	Assignment	1 microM pH 10 OceanInsight Ag 532 nm	Assignment
1714 w	ν(C=O)				
					
1653 ms	δ(NH_3_^+^) + ν_asym_(CO_2_^−^)				
					
		1627 s	δ(NH_3_^+^)	1629 m	δ(NH_2_)
1625 ms	δ(NH_3_^+^) + ν_asym_(CO_2_^−^)				
1598 ms	δ(NH_3_^+^) + ν_asym_(CO_2_^−^)	1602 s	δ(NH_3_^+^)	1597 m	ν_asym_(CO_2_^−^)
1576 ms	δ(NH_3_^+^) + ν_asym_(CO_2_^−^)			1575 m	ν_asym_(CO_2_^−^)
1551 ms	ν_asym_(CO_2_^-^)	1562 m,sh	ν_asym_(CO_2_^-^)	1556 m	ν_asym_(CO_2_^−^)
					
1532 m	ν_asym_(CO_2_^−^) second site				
1513 s	δ(NH_3_^+^) + ν_asym_(CO_2_^−^)				
1467 s	δ(CH_2_) scissoring	1474 s	δ(CH_2_) scissoring	1463 s	δ(CH_2_) scissoring
1447 m, sh	δ(CH_2_) scissoring				
		1437 m,sh	δ(CH_2_) scissoring		
1400 m	ν_sym_(CO_2_^−^)	1398 m	ν_sym_(CO_2_^−^)		
1365 s	ν_sym_(CO_2_^−^)	1362 s	ν_sym_(CO_2_^−^)	1388 mw	ν_sym_(CO_2_^−^)
1312 ms	δ(CH_2_) wagging			1301 ms	δ(CH_2_) wagging
1268 m, sh	C19-O14-H18 bend.	1264 m, sh, br	C19-O14-H18 bend.		
1239 m, sh	CH_2_ twisting			1239 ms, sh	CH_2_ twisting
1195 m, sh	CH_2_ twisting	1190 m	HCC + HNC bend.	1189 m, sh	CH_2_ twisting
1180 m	HNC bending				
1127 m	HNC bending				
1114 m,br	C19-O14-H18 bend + ν(C–C) str.				
1090 m	C6–O1 str.			1106 m	C6-O1 str.
1047 m, br	ν(C–O) str.			1060 ms	ν(C–O) str.
1002 w	HCC bend.			1002 w	HCC bend.
		989 w	HNC bending		
928 mw	ν(C–C) str.	934 ms	ν(C–C) str.	923 mw	ν(C–C) str.
915 mw	ν(C–C) str.	903 w	ν(N-C) str. +HNC bend.		ν(C–C) str.
		867 w	ν(C–C) str.		
855 m, br	ν(C–C) str.			853 ms	ν(C–C) str.
822 mw,sh	ν(C–C) str	835 w	O10=C19 out of plane	823 m	ν(C–C) str.
778 m	δ(CH_2_) rocking				
768 m	δ(CH_2_) rocking	768 m	δ(CH_2_) rocking	772 vw	δ(CH_2_) rocking
		756 m	δ(CH_2_) rocking		
680 m	δ(CO_2_^−^) bending	681 m	δ(CO_2_^−^) bending	678 s	δ(CO_2_^−^) bending
		652 m, sh			
		628 m	O7=C6–O1 bending		
612 s	O7=C6–O1 bending			613 w	O7=C6–O1 bending
566 w	O10=C19–O14 bending	555 w	O10=C19–O14 bending		
522 w	Water libration	527 w			
483 w	O7=C6–C8 bending	485 w	O7=C6–C8 bending	487 mw	O7=C6–C8 bending
		450 ms			
		439 ms			
405 w	N2-C8-C6 bending	406 w	N2-C8-C6 bending	412 w	N2-C8-C6 bending
		352 w	skeletal mode		
					
236 vs	Ag ··· O1 stretching	225 vs	Au ··· N2 stretching	236 vs	Ag ··· O1 stretching

The 1625 cm^−1^ band of Glu at pH 3.3 is assigned to the δ(NH_3_^+^) + ν_asym_(COO^−^) mode based on the calculated value of 1605 cm^−1^ for Glu-H_2_O, while the 1615 cm^−1^ band observed in SERS spectrum of Glu at pH 12 is assigned to the NH_2_ scissoring mode based on the normal modes of Na-glutamate (Table S5 in the Supplementary Information). Binding of Glu to gold has the effect of enhancing the bands corresponding to δ(NH_3_^+^) modes observed at 1602 and 1627 cm^−1^ (Figure 4 and Table 3).

The reason for selective amplification of the SERS band intensities is explained in Figure 3 of Yamamoto and Itoh [70]. The most amplified part of the SERS spectrum is the one that coincides with the plasmonic resonance energy of the metal nanoparticles of the substrate; this parameter changes from colloids to solid substrates and differs to a lesser extent for different hotspots on irregular surfaces of a given solid substrate. The electric field created by the substrate and surrounding molecules can have a great effect on the number of bands appearing in a SERS spectrum, as Aranda et al. demonstrated in the case of pyridine (Figure 4 in ref. [18]).

To clarify the nature of the binding of Glu to nanostructured Ag and Au metal surfaces, DLS experiments were performed, which yielded similar particle sizes both for gold nanoparticles in the colloid, in mixtures with water, and in mixtures of 1 mM glutamic acid at pH 3.5 and pH 12 (Table 4). In all cases, it was found that particles were negatively charged at the slipping layer, and their zeta potential was found to be lower than −30 mV, indicating that particles were stable [71].

**Figure 8:**
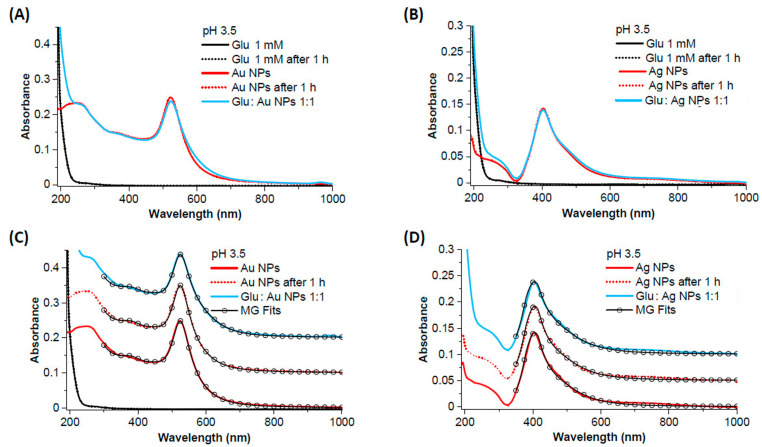
The UV-VIS spectra of Glu 1 mM, Au NPs (**left**), and Ag NPs (**right**) at pH 3.5. (**A**) Comparison of the UV-VIS spectra of Glu, bare Au, and the 1:1 mixture of Glu 1 mM and Au NPs. (**B**) The comparison of the UV-VIS spectra of Glu, bare Ag, and the 1:1 mixture of Glu 1 mM and Ag NPs. The decrease in intensity of the plasmon peak and the increase in absorbance in the red region are observed in the spectra of the mixtures in (**A**,**B**). (**C**,**D**) Mie–Gans fit (open circles) of the spectra in (**A**,**B**), showing that the proportion of non-spherical nanoparticles increases in the mixtures of Glu with NPs (39% for Au and 72% for Ag) compared to the bare NPs (24% for Au and 69% for Ag) due to the aggregation of the NPs. The spectra have been shifted for clarity.

The UV-VIS spectra of Glu 1 mM, Au NPs, Ag NPs, and the 1:1 mixture of NPs and Glu at pH 3.5 are shown in Figure 8A–D. The spectra of Glu exhibit an absorption edge at wavelengths below 300 nm, while the absorption spectra of the aqueous solutions of Au and Ag NPs show only the surface plasmon bands expected for spherical nanoparticles, centered at 523 nm for Au and 403 nm for Ag, respectively, as well as the shoulders due to interband transitions at shorter wavelengths (Figure 8A,B) [72]. As is typical for pure metal NPs obtained by laser ablation in water, there are no other absorption bands in the UV region, such as the bands due to organic stabilizers or synthesis by-products of metal NPs obtained by chemical methods [73]. The spectra of Au- and Ag-NP remain unchanged even after 1 h in the aqueous solution. However, when the Glu solution is added to the Au and Ag NPs, a change in the surface plasmon band can be observed (Figure 8A,B), which consists of a decrease in peak intensity and an increase in plasmon absorption in the red spectral region. Fitting the experimental spectra with a code based on the Mie theory for spherical nanoparticles and the Gans model for non-spherical particles (MG fit) was performed [72]. Figure 8C,D shows that these spectral changes are due to the increase in the proportion of non-spherical particles (i.e., aggregates of NPs) in the mixture (39% for Au and 72% for Ag) compared to the bare NPs (24% for Au and 69% for Ag).

**Figure 9:**
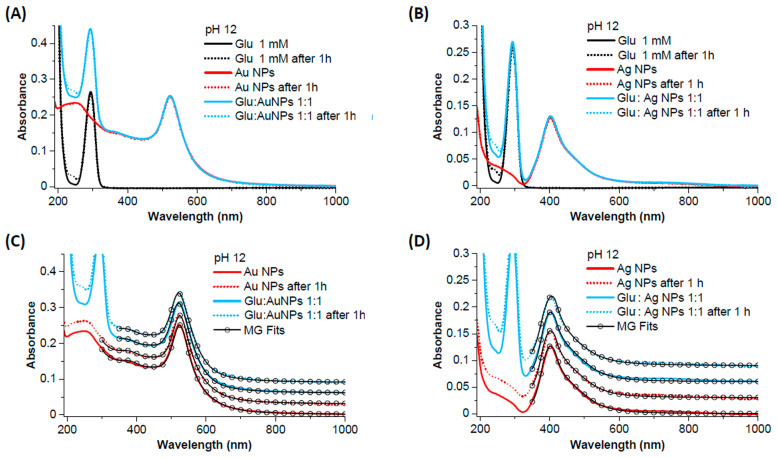
UV–VIS spectra of Glu 1 mM, Au NPs (**left**), and Ag NPs (**right**) at pH 12. (**A**) Comparison of the UV–VIS spectra of Glu, bare Au, and the 1:1 mixture of Glu 1 mM and Au NPs. (**B**) The comparison of the UV–VIS spectra of Glu, bare Ag, and the 1:1 mixture of Glu 1 mM and Ag NPs. (**C**,**D**) Mie–Gans fit (open circles) of the spectra in (**A**,**B**), indicating that the proportion of non-spherical nanoparticles in the mixtures of Glu with NPs even after 1 h (27% for Au and 69% for Ag) corresponds to that of the bare NPs (27% for Au and 70% for Ag), that is, that the aggregation of the particles does not increase due to the interaction with Glu. The spectra have been shifted for clarity.

The UV-VIS spectra of Glu 1 mM, Au NPs, Ag NPs, and the 1:1 mixtures of NPs and Glu at pH 12 are shown in Figure 9A–D. A peak at 293 nm appears in the spectra of the Glu-containing solutions, which was not observed at pH 3.5 (Figure 8A,B). The peak that would correspond to sodium glutamate is expected at 210 nm [74]; therefore, the peak we observed could correspond to the multiple aggregates of the buffering agent NaOH with Glu. After 1 h, the absorbance of the Glu-containing solutions continued to develop with an increase in the 200–250 nm range, and this absorbance is assigned to monosodium glutamate [74]. Instead, the spectra of the bare Au and Ag NPs remain unchanged even after 1 h in the aqueous solution. After mixing with Glu and ageing for 1 h, no changes are observed in the absorbance of the NPs compared to the unmixed NPs. The absorbance of Glu is also equivalent to that of the compound alone, as it shows the same change in the 200–250 nm range after one hour of ageing. The fitting of the experimental spectra (Figure 9C,D) confirmed that no changes occurred and that mixing or ageing did not lead to aggregation of the NPs.

**Figure 10:**
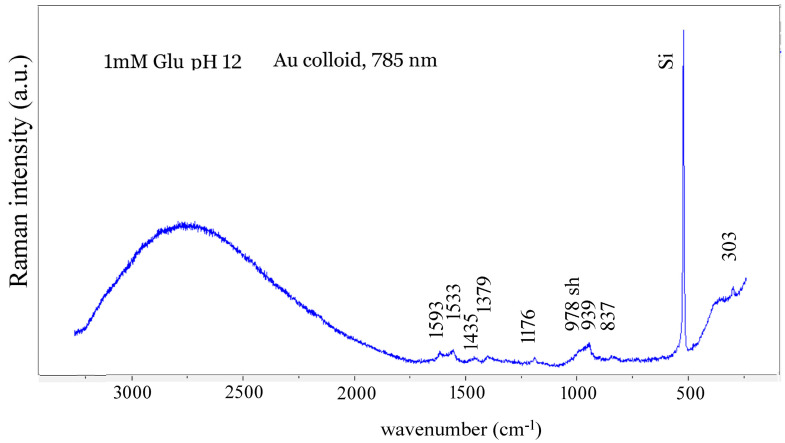
SERS spectrum of 1 mM solution of L-Glu at pH 12 obtained when mixed 1:1 *v*/*v* with gold colloid and dropped on Si plate. Excitation line: 785 nm.

The SERS spectrum of 1 mM Glu at pH 12 obtained with the Au colloid shows no discernible bands above 1600 cm^−1^ due to a huge, very broad band centered around 2700 cm^−1^, which is attributed to photoluminescence of silicon under a 785 nm laser beam (Figure 10). The observed bands are as follows: 1593 cm^−1^—assigned to δ(NH_2_), 1533 cm^−1^—assigned to ν_asym_(COO^−^), 1435 cm^−1^—assigned to δ(CH_2_) bending, 1379 cm^−1^—assigned to ν_sym_(COO^−^), and the three bands at 837, 939, and 978 cm^−1^ to ν(C–C) stretching vibrations. Some of the bands are close to the ones observed in SERS spectra of Glu with Au colloids by Philip, like the 1365 cm^−1^ band she assigned to the CH_2_ wag but is assigned to the CO_2_^-^ symmetric stretching (Table 3).

**Figure 11:**
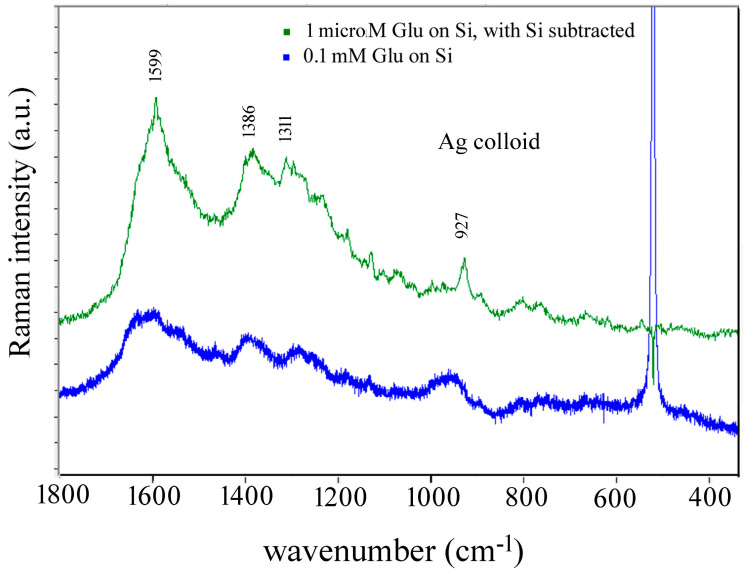
Comparison of SERS spectra of 0.1 μM Glu solution at pH 3.5 with the SERS spectrum of 0.1 mM Glu at pH 3.5, both obtained using Ag colloid and 532 nm laser.

Overlapping of bands is also observed in SERS spectra of Glu obtained with silver colloids (Figure 11). The observed band at 927 cm^−1^ can be compared with the 930 cm^−1^ band reported by Stewart and Fredericks [45].

The discrimination of amino acids by SERS can be challenging. Sengupta et al. [51] used μM concentrations of alanine, lysine, and glutamic acid and obtained very similar spectra with the strongest bands at 1640, 1401, and 1379 cm^−1^. The SERS spectra of Glu and Asp acids, also mixed in μM concentrations with colloids, differed mainly in the C–C stretching vibration as follows: the one at 830 cm^−1^ was characteristic of Glu, while that of Asp acid was at 785 cm^−1^, as reported by O’Neal [5]. O’Neal [5] and Chumanov [44] observed the band at 830 cm^−1^ assigned to the C–C stretching, while we assigned the 855 and 822 cm^−1^ bands to two C-C stretching modes at pH 3.3 and to 853 and 823 cm^−1^ bands at pH 10 (Table 3 and Figure 4 and Figure 7). Guicheteau et al. [52] reported SERS spectra of dried colloidal 6.8 mM Glu solutions having characteristic bands at 814, 946, 1236, 1397, 1549, and 1599 cm^−1^, which are consistent with the bands we observed at 1239, 1393, 1542, and 1596 cm^−1^ in the SERS spectrum of Glu at pH 3.5 obtained with silver colloid.

Our comparison of calculated normal modes of Glu-Au and Glu-Ag with observed SERS bands is limited by the fact that models of Glu-metal complexes are not zwitterions, while in experimental conditions, Glu is always zwitterionic (scheme in Figure 3). However, we conclude that binding of silver increases the intensity of the 680 cm^−1^ band attributed to O=C-O bending mode, while binding of gold increases the intensity of the 458 cm^−1^ band assigned to O=C-C bending mode.

## 4. Conclusions

We report an experimental and computational study of the binding of glutamic acid to gold and silver substrates by comparing ab initio results of binding gold and silver atoms to a free Glu molecule with the experimental spectra of Glu solutions at pH values of 3.3, 3.5, 7, 10, and 12. The lowest Glu concentration for which SERS spectra were observed is 0.1 nM at pH 7 using Sersitive substrates and 785 nm laser line as an excitation source. Observed variability in SERS spectra of glutamic acid reflects the diversity of binding sites present in substrates used.

Binding of silver atoms causes selective amplification of the modes associated with the binding site; here the carbonyl and hydroxyl groups are closer to the amino group. The energy difference between Glu-H_2_O and Glu-Au complex is -0.087 eV in favor of binding with water at the b3lyp/lanl2dz level of theory; hence, glutamic acid is hydrated when it binds to metal atoms.

At low pH, the assignment was aided by ab initio calculations of crystal phonons, since the zwitterionic form of the Glu molecule at pH 3.5 has the same protonation state as in the crystal. At basic pH, the ab initio calculation of the normal modes of the sodium glutamate molecule provided the basis for the assignment. Analysis of the UV–VIS spectra of Ag and Au colloids and their solutions with glutamic acid at pH 12 revealed that no aggregation of the metal particles occurs. Therefore, the width of the spectral bands is primarily attributed to different types of Glu-metal binding.

## Figures and Tables

**Table 1:** Comparison of selected scaled calculated vibrational modes of neutral glutamic acid, glutamic acid monohydrate, and glutamic acid bound to Au and Ag in the interval 1300–1780 cm^−^^1^. A complete list of calculated modes with potential energy distribution is given in Supporting Information, Tables S1–S4.

Calculated Vibrations of Neutral Form of L-Glutamic Acid (Scaled by 0.968)		Calculated Vibrations of Glutamic Acid Monohydrate (Scaled by 0.968)		Calculated Vibrations of Glutamic Acid Bound to Au (Scaled by 0.968)		Calculated Vibrations of Glutamic Acid Bound to Ag (Scaled by 0.968)	
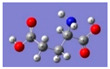 Normal modes description		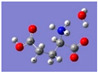 Normal modes description		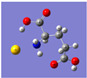 Normal modes description		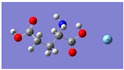 Normal modes description	
		1717	ν(C=O)				
1691	ν(C_6_=O_7_)	1701	ν_asym_(CO_2_^-^)			1694	ν(C=O)
		1659	δ(NH_3_^+^) bend. + ν_asym_(CO_2_^-^)	1663	ν(C_6_ =O_7_)		
1644	δ(NH_2_) scissor.			1640	ν(C_19_=O_10_) + δ(NH_2_) scissoring	1644	δ(NH_2_) scissor.
1633	ν(C_19_=O_10_)					1634	ν(C=O) + δ(NH_2_) scissor
		1595	δ(NH_3_^+^) bend.	1605	δ(NH_2_) scissoring + ν(C_19_ =O_10_)		
		1560	δ(H_2_O) bending				
		1474	δ(HNC) bending				
1467	δ(CH_2_) scissoring			1465	δ(CH_2_) scissoring	1468	δ(CH_2_) scissoring
				1461	δ(CH_2_) scissoring		
1457	δ(CH_2_) scissoring	1455	δ(CH_2_) scissoring			1457	δ(CH_2_) scissoring
		1443	δ(CH_2_) scissoring				
		1379	δ(HCC) + ν(C-O)	1379	δ(CH_2_) wagging	1376	δ(CH_2_) wag.
1374	δ(COH) + ν(C-O)						
	δ(COH)			1365	δ(HCC) bending		
1357	δ(HCC) bending	1352	δ(HCC) bend.			1357	δ(HCC) bending
1323	C6-01-H3 bend.						
1315	δ(CH_2_) wagging	1316	δ(CH_2_) twisting	1319	δ(HCC) bending	1324	δ(COH) bend.
**~**							~
						676	O7=C6 out of pl.
		640	O14-H18 torsion	632	O14-H18 torsion		
621	O7=C6 out of plane	615	O7=C6-O1 bend.	611	O7=C6-O1 bend.	623	O14-H18 torsion
561	O7=C6-O1 bend.	554	O10=C19-O14 bend.	555	O7=C6-O1 bend.+ O7=C6 out of plane	561	O7=C6-O1 bend.
526	O10=C19-O14 bend.	529	Water libration	532	O10=C19-O14 bend	524	O10=C19-O14 bend
504	O7=C6-C8 bend					507	O7=C6-C8 bend
		493	O7=C6-C8 bend.				
				476	O7=C6-C8 bend.		
422	O10=C19-O14 bend	426	O10=C19-O14 bend	429	O10=C19-C15 bend.	425	O10=C19-C15 bend.
401	N-C8-C11 bend	413	N-C8-C11 bend	407	N-C8-C11 bend	403	Torsion N2-C8

**Table 2:** Comparison of selected calculated vibrational modes of glutamic acid in zwitterionic form as found in crystal with vibrations of sodium glutamate in the interval 1350–1780 cm^−1^. A complete list of calculated modes with potential energy distribution among modes is given in Supplementary Information (output of the CRYSTAL09 program, Table S5).

Calculated Vibrations of L-Glutamic Acid Crystal				Observed Bands L-Glutamic Acid Powder			Sodium Glutamate Molecule	
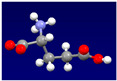				Raman infrared observed		Crystal modes description	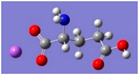 Normal mode description	
1724,	1734,	1735,	1735	1732		ν(C=O) + δ(NH_3_^+^)	1753	ν(C=O)
A	B_1_	B_2_	B_3_					
1701,	1701,	1707,	1707		1664	δ(H-N-H) + ν_asym_(COO^-^)		
A	B_3_	B_2_	B_1_					
1687,	1690,	1690,	1692	1658	1645	δ(NH_3_^+^)		
B_1_	A	B_3_	B_2_					
1626,	1627,	1640,	1649	1632, 1616	1615	ν_asym_(COO^−^) + δ(NH_3_^+^)	1626	δ(NH_2_) scissoring
A	B_3_	B_1_	B_2_					
1570,	1571,	1580,	1582	1579		δ_sym_(NH_3_^+^)		
A	B_3_	B_1_	B_2_					
1531,	1532,	1534,	1538		1509	δ(C-O-H)	1539	ν_asym_ (COO^-^)
A	B_2_	B_3_	B_1_					
1492,	1494,	1495,	1496	1512		δ(CH_2_) scissoring		
B_2_	A	B_1_	B_3_					
1458,	1466,	1468,	1477	1452		δ(CH_2_)	1437	δ(CH_2_) scissoring
B_3_	B_3_	B_1_	B_2_					
1452,	1453,	1453,	1456					
A	B_2_	A	B_1_					
1419,	1420,	1426,	1430	1441	1424	δ(CH_2_) wag + δ(COH)		
B_3_	B_1_	A	B_2_					
1411,	1411,	1415,	1416		1411	δ(CH_2_) wag + δ(HNC)	1408	δ(CH_2_) scissoring
A	B_2_	B_1_	B_3_					
1389,	1393,	1393,	1398	1409		ν_sym_(COO^−^) + δ(HCC)	1392	ν_sym_(COO^-^)
A	B_3_	B_1_	B_2_					
				1376	1376		1363	δ(HCC) bending
1349,	1349,	1352,	1356	1352	1352	δ(CH_2_) twist + δ(NH_3_^+^) rock	1347	δ(HCC) bending
B_2_	A	B_1_	B_3_					

**Table 4:** Hydrodynamic diameters and zeta potential values of Au colloid samples.

Sample	Peak 1/nm	Population 1 (%)	Peak 2/nm	Population 2 (%)	Zeta Potential/mV
Pure Au colloid	19.5 ± 0.5	99.8	73.9 ± 3.2	0.2	−39.81 ± 1.96
Au + H_2_O 1:1	15.8 ± 1.6	99.9	70.6 ± 2.7	0.1	−42.2 ± 2.7
1 mM Glu pH = 12	8.67 ± 1.3	100.0			−36.98 ± 2.55
Au + 1 mM Glu 1:1, pH = 12	13.6 ± 1.3	100.0			−41.2 ± 0.4
Au+1mM Glu 1:1, pH = 3.5	14.9 ± 1.0	100.0			−37.6 ± 2.8

## Data Availability

Data are available upon request from authors.

## Supplementary Materials

The following supporting information can be downloaded at: https://www.mdpi.com/article/10.3390/bios14110522/s1, Figure S1: Transmission electron microscopy estimates the size of particles in silver colloid to be 26 ± 6 nm; Figure S2: Transmission electron microscopy estimates the size of particles in gold colloid to be 15.5 ± 3.9 nm. Figure S3: Comparison of SERS spectra of Glu at pH 12 for 10^-4^ M and 10^-9^ M concentrations using OceanInsight substrate and 532 nm excitation. Table S1: Potential energy distribution among normal modes of glutamic acid with the list of internal coordinates; Table S2: Potential energy distribution among normal modes of glutamic acid monohydrate with the list of internal coordinates; Table S3: Potential energy distribution among normal modes of glutamic acid bound to a gold atom with the list of internal coordinates; Table S4: Potential energy distribution among normal modes of glutamic acid bound to a silver atom with the list of internal coordinates; Table S5: Potential energy distribution among normal modes of sodium glutamate with the list of internal coordinates.
