# Supplementary material for: Binding of Glutamic Acid to Silver and Gold Nanoparticles Investigated by Surface-Enhanced Raman Spectroscopy
Source: Biosensors (Basel). 2024 Oct 25;14(11):522. doi: 10.3390/bios14110522 (PMC11591887; doi:10.3390/bios14110522)
Supplement: Supplementary file 1 [file biosensors-14-00522-s001.zip › Supplementary_information_SERS_Glu_Biosensors.pdf]

Supplementary information for the article:

„Binding of glutamic acid to silver and gold nanoparticles investigated by surface-enhanced Raman spectroscopy“,

by V. Mohaček-Grošev, M. Škrabić, H. Gebavi, V. Blažek Bregović, I. Marić, V. Amendola and J. Grdadolnik

prepared for Biosensors

1. The output of phonon calculation using CRYSTAL09 program (separate file: glutaminskafr_2023.out) for inspection online via website: https://crysplot.crystalsolutions.eu/web_pages_yves3/vibration.html

2. Supplementary figures S1, S2, S3

3. Supplementary Table S1: Potential energy distrubution among normal modes of glutamic acid with the list of internal coordinates

4. Supplementary Table S2: Potential energy distrubution among normal modes of glutamic acid monohydrate with the list of internal coordinates

5. Supplementary Table S3: Potential energy distrubution among normal modes of glutamic acid bound to a gold atom with the list of internal coordinates

6. Supplementary Table S4: Potential energy distrubution among normal modes of glutamic acid bound to a silver atom with the list of internal coordinates

7. Supplementary Table S5: Potential energy distrubution among normal modes of sodium glutamate with the list of internal coordinates

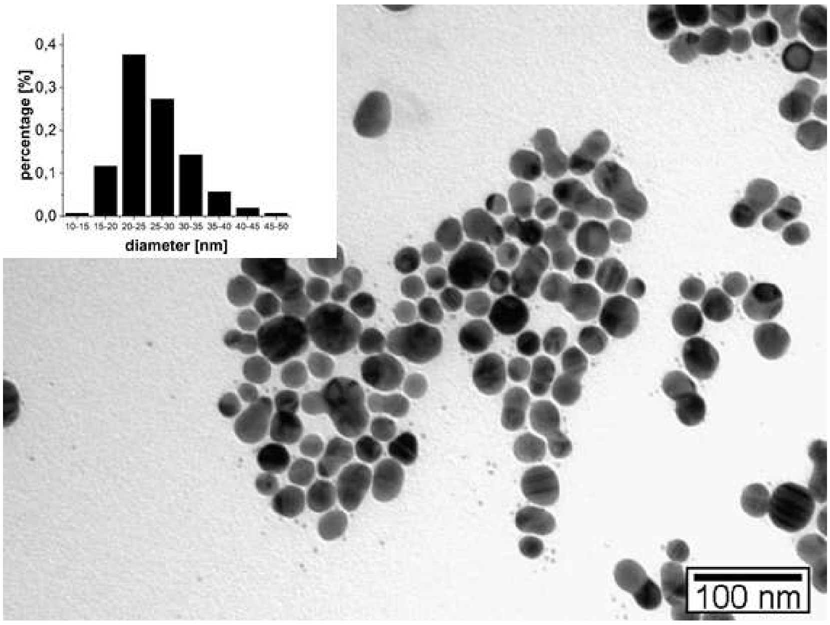

Supplementary Figure S1. Transmission electron microscopy estimates the size of particles in silver colloid to be 26 ± 6 nm.

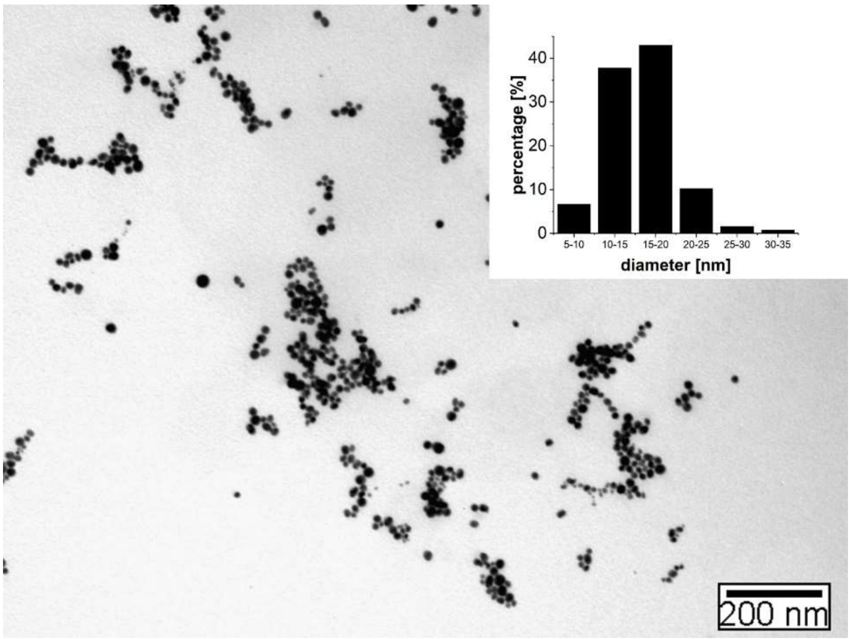

Supplementary Figure S2. Transmission electron microscopy estimates the size of particles in gold colloid to be 15.5 ± 3.9 nm.

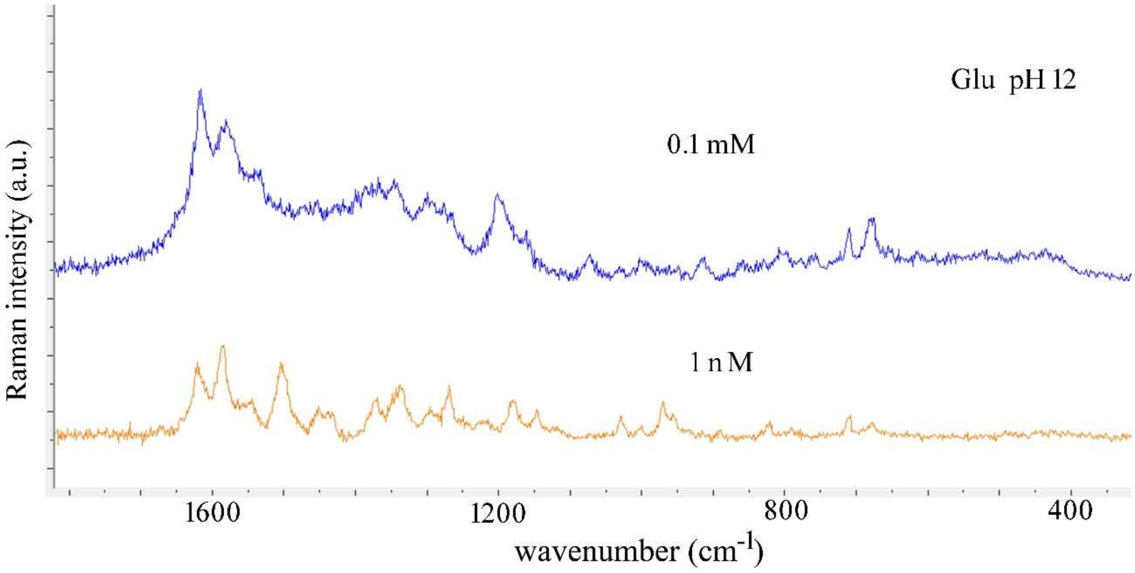

Supplementary Figure S3. Comparison of SERS spectra of Glu at pH 12 for 10^{-4} M and 10^{-9} M concentrations using OceanInsight substrate and 532 nm excitation.

Supplementary Table S1. Potential energy distribution among normal modes of glutamic acid calculated using BALGA program (G. Keresztury, G. Jalsovszky, J. Mol. Struct. 10 (1971) 304-305.; H. Rostkowski, L. Lapinski, M. Nowak, Vib. Spectrosc. 49 (2009) 43-51). List of internal coordinates is given below the table. B3LYP/lanl2dz.

Mode number	Calculated (scaled by 0.968)	PED (%)	assignment
1.	3520	86 S_4 + 12 S_2	asym. NH_2 stretch
2.	3519	87 S_2 + 12 S_4	O14-H18 stretch.
3.	3404	97 S_3	sym. NH_2 stretch.
4.	3140	99 S_1	O1-H3 stretch.
5.	3064	91 S_9	CH_2 asym. stretch.
6.	3039	84 S_7	CH_2 asym. stretch.
7.	2996	93 S_5	C8-H9 stretch
8.	2987	80 S_8	CH_2 sym. stretch
9.	2968	87 S_6	CH_2 sym. stretch
10.	1691	77 S_{10}	C6=O7 stretch.
11.	1644	56 S_{42} + 20 S_{28}	NH2 scissoring
12.	1633	60S_{11} + 17 S_{42}	C19=O10 stretch.
13.	1467	49 S_{33} + 43 S_{35}	CH_2 scissoring
14.	1457	64 S_{39} + 27 S_{37}	CH_2 scissoring
15.	1374	20 S_{37} + 20 S_{33} + 14 S_{35}	CH_2 bend.
16.	1357	39 S_{32} + 26 S_{31} +17 S_{29} + 11 S_{26}	H9-C6-C8 bend.+...
17.	1323	38 S_{19} + 12 S_{31}	C6-O1-H3 bend.
18.	1315	25 S_{37} + 15 S_{35} + 12 S_{36}	CH_2 wagg.
19.	1305	23 S_{19} + 13 S_{20}	C19-O14-H18 bend.
20.	1282	23 S_{31} + 15 S_{34} +14 S_{32}	H9-C8- C11 bend.
21.	1216	26 S_{32} + 18 S_{34} + 13 S_{29}	H9-C8-C6 bend.
22.	1199	29 S_{38} + 20 S_{40} + 18 S_{20}	CH_2 twist.
23.	1155	27 S_{12} + 16 S_{14} + 13 S_{29}	C6-O1 stretch.
24.	1133	22 S_{36} + 16 S_{29} + 14 S_{20} + 10 S_{40}	CH_2 twist + HNC bend.
25.	1116	26 S_{15} + 17 S_{23} + 17 S_{18}	C8-C11 stretch. + O7=C6-C8 bend.
26.	1059	23 S_{13} + 22 S_{20} + 12 S_{38} + 11 S_{17}	C19-O14 stretch. + ...
27.	1047	27 S_{16} + 13 S_{15} + 12 S_{29} + 11 S_{12}	C11-C15 stretch. + ..
28.	971	24 S_{16} + 10 S_{17} + 10 S_{36}	C-C stretch. + ..
29.	948	17 S_{34} + 15 S_{40} + 13 S_{18} + 10 S_{13}	CH_2 rock.
30.	927	86 S_{43}	O1-H3 torsion
31.	905	15 S_{16} + 10 S_{12} + ..	C-C stretch.
32.	806	28 S_{28} + 14 S_{14} + ..	NH_2wagg.+..
33.	798	21 S_{28} + 14 S_{18} + 12 S_{17}	NH2wagg + N2-C8 stretch.
34.	751	15 S_{36} + 10 S_{17} + ...	CH2 rock + C15-C19 stretch
35.	713	32 S_{51} + 21 S_{49} + 10 S_{13}	O10=C19 out of pl.
36.	678	34 S_{50} + 19 S_{21}	O7=C6 out of pl.+CH_2 bend.

Table S1 continued

Mode number	Calculated (scaled by 0.968)	PED (%)	assignment
37.	621	71 S_{49}	O14-H18 torsion
38.	561	40 S_{21} + 15 S_{50} +12 S_{41}	O7=C6-O1 bend
39.	526	59 S_{22} + 12 S_{17}	O10=C19-O14 bend.
40.	504	43 S_{23} + 22 S_{25}	C8-C6=O7 bend
41.	422	37 S_{24} + ...	C15-C19=O10 bend.
42.	401	35 S_{46} + 33 S_{30}	torsion around N2-C8+N2-C8-C11 bend
43.	387	40 S_{46} + 21 S_{30} + ...	torsion around N2-C8
44.	337	22 S_{25} + 21 S_{21}	skeletal mode
45.	303	24 S_{25} + 16 S_{41} + 13 S_{24} + 10 S_{27}	N2-C8-C6 bend.+..
46.	199	34 S_{27} + 32 S_{26}	skeletal mode
47.	159	18 S_{26} + 15 S_{27} + 12 S_{47} + 11 S_{45}	C6-C8-C11 bend.
48.	108	47 S_{47} + 24 S_{44}	C8-C11-C15-C19 torsion
49.	82	47 S_{44} + 32 S_{45}	torsion around C6-C8
50.	43	46 S_{45} + 24 S_{47} + 21 S_{44}	torsion around C8-C11+torsion around C11-C15
51.	34	77 S_{48}	torsion around C15-C19

Definition of internal coordinates of glutamic acid

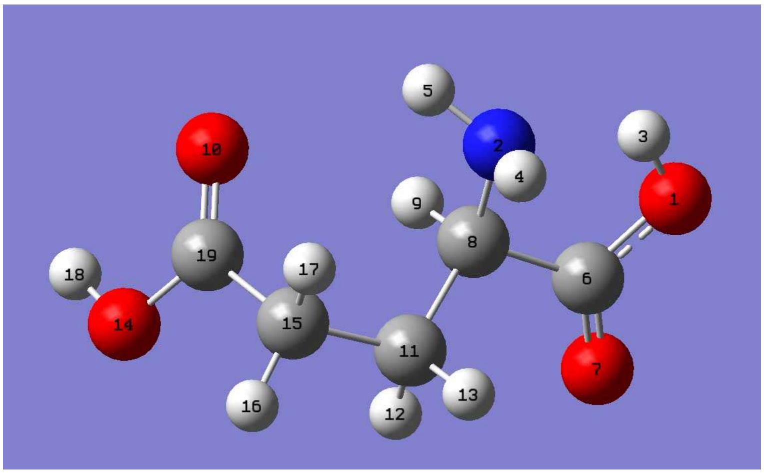

1. $S_1 = \Delta r(O1\text{-}H3)$

2. $S_2 = \Delta r(O14\text{-}H18)$

3. $S_3 = \Delta r(N2\text{-}H4) + \Delta r(N2\text{-}H5)$

4. $S_4 = \Delta r(N2\text{-}H4) - \Delta r(N2\text{-}H5)$

5. $S_5 = \Delta r(C8\text{-}H9)$

6. $S_6 = \Delta r(C11\text{-}H12) + \Delta r(C11\text{-}H13)$

7. $S_7 = \Delta r(C11\text{-}H12) - \Delta r(C11\text{-}H13)$

8. $S_8 = \Delta r(C15\text{-}H16) + \Delta r(C15\text{-}H17)$

9. $S_9 = \Delta r(C15\text{-}H16) - \Delta r(C15\text{-}H17)$

10. $S_{10} = \Delta r(C6{=}O7)$

11. $S_{11} = \Delta r(C19{=}O10)$

12. $S_{12} = \Delta r(C6\text{-}O1)$

13. $S_{13} = \Delta r(C19\text{-}O14)$

14. $S_{14} = \Delta r(C6\text{-}C8)$

15. $S_{15} = \Delta r(C8\text{-}C11)$

16 $S_{16} = \Delta r(C11\text{-}C15)$

17. $S_{17} = \Delta r(C15\text{-}C19)$

18. $S_{18} = \Delta r(C8\text{-}N2)$

19. $S_{19} = \Delta\theta(C6\text{-}O1\text{-}H3)$

20. $S_{20} = \Delta\theta(C19\text{-}O14\text{-}H18)$

21. $S_{21} = \Delta\theta(O7{=}C6\text{-}O1)$

22. $S_{22} = \Delta\theta(O10{=}C19\text{-}O14)$

23. $S_{23} = \Delta\theta(O7{=}C6\text{-}C8)$

24. $S_{24} = \Delta\theta(O10{=}C19\text{-}C15)$

25. $S_{25} = \Delta\theta(N2\text{-}C8\text{-}C6)$

26. $S_{26} = \Delta\theta(C6\text{-}C8\text{-}C11)$

27. $S_{27} = \Delta\theta(C11\text{-}C15\text{-}C19)$

28. $S_{28} = \Delta\theta(H4\text{-}N2\text{-}C8) + \Delta\theta(H5\text{-}N2\text{-}C8)$

29. $S_{29} = \Delta\theta(H4\text{-}N2\text{-}C8) - \Delta\theta(H5\text{-}N2\text{-}C8)$

30. $S_{30} = \Delta\theta(N2\text{-}C8\text{-}C11)$

31. $S_{31} = \Delta\theta(H9\text{-}C8\text{-}C11)$

32. $S_{32} = \Delta\theta(H9\text{-}C8\text{-}C6)$

33. $S_{33} = \Delta\theta(H12\text{-}C11\text{-}C8) + \Delta\theta(H13\text{-}C11\text{-}C8)$

34. $S_{34} = \Delta\theta(H12\text{-}C11\text{-}C8) - \Delta\theta(H13\text{-}C11\text{-}C8)$

35. $S_{35} = \Delta\theta(H12\text{-}C11\text{-}C15) + \Delta\theta(H13\text{-}C11\text{-}C15)$

36. $S_{36} = \Delta\theta(H12\text{-}C11\text{-}C15) - \Delta\theta(H13\text{-}C11\text{-}C15)$

37. $S_{37} = \Delta\theta(H16\text{-}C15\text{-}C11) + \Delta\theta(H17\text{-}C15\text{-}C11)$

38. $S_{38} = \Delta\theta(H16\text{-}C15\text{-}C11) - \Delta\theta(H17\text{-}C15\text{-}C11)$

39. $S_{39} = \Delta\theta(H16\text{-}C15\text{-}C19) + \Delta\theta(H17\text{-}C15\text{-}C19)$

40. $S_{40} = \Delta\theta(H16\text{-}C15\text{-}C19) - \Delta\theta(H17\text{-}C15\text{-}C19)$

41. $S_{41} = \Delta\theta(C8\text{-}C11\text{-}C15)$

42. $S_{42} = \Delta\theta(H4\text{-}N2\text{-}H5)$

43. $S_{43} = \Delta\tau(H3\text{-}O1\text{-}C6{=}O7)$

44. $S_{44} = \Delta\tau(O1\text{-}C6\text{-}C8\text{-}C11) + \Delta\tau(O7{=}C6\text{-}C8\text{-}C11) + \Delta\tau(O1\text{-}C6\text{-}C8\text{-}N2) + \Delta\tau(N2\text{-}C8\text{-}C6{=}O7) + \Delta\tau(H9\text{-}C8\text{-}C6{=}O7)$

45. $S_{45} = \Delta\tau(N2\text{-}C8\text{-}C11\text{-}C15) + \Delta\tau(H9\text{-}C8\text{-}C11\text{-}C15) + \Delta\tau(C15\text{-}C11\text{-}C8\text{-}C6) + \Delta\tau(H12\text{-}C11\text{-}C8\text{-}C6) + \Delta\tau(H13\text{-}C11\text{-}C8\text{-}C6)$

46. $S_{46} = \Delta\tau(H4\text{-}N2\text{-}C8\text{-}C11) + \Delta\tau(H5\text{-}N2\text{-}C8\text{-}C11) + \Delta\tau(H4\text{-}N2\text{-}C8\text{-}C6) + \Delta\tau(H5\text{-}N2\text{-}C8\text{-}C6)$

47. $S_{47} = \Delta\tau(C8\text{-}C11\text{-}C15\text{-}C19)$

48. $S_{48} = \Delta\tau(H16\text{-}C15\text{-}C19{=}O10) + \Delta\tau(H17\text{-}C15\text{-}C19{=}O10)$

49. $S_{49} = \Delta\tau(H18\text{-}O14\text{-}C19{=}O10)$

50. $S_{50} = \Delta\lambda(O7{=}C6)$ out of plane

51. $S_{51} = \Delta\lambda(O10{=}C19)$ out of plane

Supplementary Table S2. Potential energy distribution among normal modes of glutamic acid monohydrate calculated using BALGA program(G. Keresztury, G. Jalsovszky, J. Mol. Struct. 10 (1971) 304-305.; H. Rostkowski, L. Lapinski, M. Nowak, Vib. Spectrosc. 49 (2009) 43-51). List of internal coordinates is given below the table. B3LYP/6-311++G(d,p).

Mode number	Calculated (scaled by 0.968)	PED (%)	assignment
1.	3762	100 S_{52}	O20-H21 stretch.
2.	3616	100 S_1	O14-H18 stretch.
3.	3394	75 S_4 + 16 S_2 + 10 S_3	asym. NH_3^+ stretch.
4.	3238	44 S_2 + 31 S_3 + 25 S_4	sym. NH_3^+ stretch.
5.	3130	95 S_{53}	O20-H22 stretch .
6.	3039	55 S_7 + 28 S_9 + 14 S_6	CH_2 asym. stretch.
7.	3027	58 S_9 + 23 S_7 + 13 S_8	CH_2 asym. stretch.
8.	3005	95 S_5	C8-H9 stretch.
9.	2968	66 S_8 + 20 S_6 + 12 S_9	CH_2 sym. stretch.
10.	2959	58 S_6 + 19 S_7 + 19 S_8	CH_2 sym. stretch.
11.	2878	60 S_3 + 38 S_2	asym. NH_3^+ stretch.
12.	1717	67 S_{10} + 11 S_{11}	C19=O10 stretch.
13.	1701	55 S_{11} + 14 S_{10} + 14 S_{42}	C6-O1+C6-O7 stretch.
14.	1659	41 S_{42} + 25 S_{43} + 25 S_{11}	NH_3^+ bend. + asym CO_2 stretch.
15.	1595	48 S_{43} + 26 S_{42}	NH_3^+ bend.
16.	1560	80 S_{54}	H_2O bend.
17.	1474	76 S_{27}	H-N-C bend.
18.	1455	42 S_{39} + 18 S_{35} + 16 S_{33} + 16 S_{37}	CH_2 scissor.
19.	1443	31 S_{33} + 28 S_{35} + 18 S_{39} + 13 S_{37}	CH_2 scissor.
20.	1379	19 S_{33} + 16 S_{37} + 12 S_{13} + 10 S_{31}	H-C-C bend.+ C19-O14 stretch
21.	1352	52 S_{31} + 10 S_{32}	H9-C11-C8 bend.
22.	1316	23 S_{35} + 14 S_{32} + 13 S_{33} + 10 S_{19}	CH_2 wagg.
23.	1297	28 S_{37} + 16 S_{36} +14 S_{39}	CH_2 twist.
24.	1294	66 S_{12}	sym. CO_2 stretch.
25.	1260	21 S_{34} + 17 S_{32} + 10 S_{31}	CH_2 wagg + ..
26.	1213	19 S_{28} + 13 S_{34} + ...	H-N-C + H-C-C bend.
27.	1198	27 S_{19} + 18 S_{40} + 11 S_{38}	H18-O14-C19 bend.+.
28.	1144	19 S_{38} + 19 S_{13} + 18 S_{19} + ...	C19-O14 stretch.+...
29.	1120	15 S_{32} + 12 S_{28} + 11 S_{29}	HCC bend. +HNC bend.
30.	1076	36 S_{29} + 19 S_{32} + 10 S_{36}	H-N-C bend.
31.	1045	25 S_{15} + 16 S_{18} + 15 S_{40}	C-C stretch. +C-N stretch
32.	987	30 S_{16} + 13 S_{40} + ...	C11-C15 stretch. + ..
33.	949	19 S_{34} + 14 S_{16} + ...	C6-C8 stretch. + ..
34.	890	35 S_{18} + 16 S_{38} + 10 S_{40}	CH_2 rock.

Table S2. continued

Mode number	Calculated (scaled by 0.968)	PED (%)	assignment
35.	871	37 S_{58} + 20 S_{14}	O20-H22···O1 bend.
36.	831	44 S_{58} + 14 S_{20} + 13 S_{14}	O20-H22···O1 bend +CO_2 bend.
37.	813	18 S_{17} + 17 S_{50} +16 S_{58}	C15-C19 stretch.
38.	761	23 S_{20} + 12 S_{18} + 11 S_{15} + ..	O7=C6=O1 bend. + C-N str.
39.	735	23 S_{51} + 20 S_{49} +14 S_{36}	O10=C19 out of plane bend.
40.	717	35 S_{50} + 16 S_{20} + ..	O7=C6 out of pl.
41.	640	49 S_{49}	torsion O18-H14
42.	615	20 S_{20} + 19 S_{49} + 10 S_{50} + 10 S_{14}	O7=C6=O1 bend. +O18-H14 torsion
43.	554	51 S_{21} + 16 S_{17} + 10 S_{51}	O10=C19-O14 bend.
44.	529	28 S_{57} + 22 S_{60} + 15 S_{46} + 12 S_{54}	H22···O1=C6-C8 torsion
45.	493	48 S_{22} + 15 S_{30} + 12 S_{18} + ..	O7=C6-C8 bend.
46.	426	26 S_{23} + 10 S_{14} +..	O10=C19-C15 bend
47.	413	27 S_{23} + 15 S_{55} + 12 S_{57} + 12 S_{46}	O10=C19-C15 bend.+ O20···H3 stretch
48.	393	25 S_{30} + 17 S_{46} + 12 S_{57}	N2-C8-C11 bend.
49.	325	17 S_{41} + 10 S_{23} + 10 S_{26} + 10 S_{30}	skeletal mode
50.	306	55 S_{59} + 18 S_{22} + 15 S_{24}	H21-O20 out of plane + O7-C6-C8 bend.+..
51.	274	39 S_{59} +32 S_{56} + 16 S_{46} + 14 S_{24}	H21-O20 out of plne
52.	241	45 S_{56} + 17 S_{25} + 16 S_{46}	H22···O1 stretch
53.	193	41 S_{26} + 13 S_{45}	C11-C15-C19 bend.
54.	178	33 S_{55} + 26 S_{56} + 25 S_{24}	O20···H3 stretch
55.	135	40 S_{47} + 19 S_{41} + 15 S_{25} + 11 S_{44}	C8-C11-C15-C19 torsion
56.	129	41 S_{47} + 20 S_{55} + 15 S_{41}	torsion around C11-C15 + O20···H3 stretch.
57.	87	45 S_{45} + 37 S_{48}	torsion around C8-C11
58.	57	35 S_{45} + 16 S_{48} + 15 S_{44}	skeletal mode
59.	51	38 S_{57} + 23 S_{44} + 12 S_{48}	torsion H22··O1=C6-C8
60.	43	48 S_{44} + 21 S_{47} + 19 S_{48}	torsion around C6-C8

Definition of internal coordinates of glutamic acid boud with water molecule

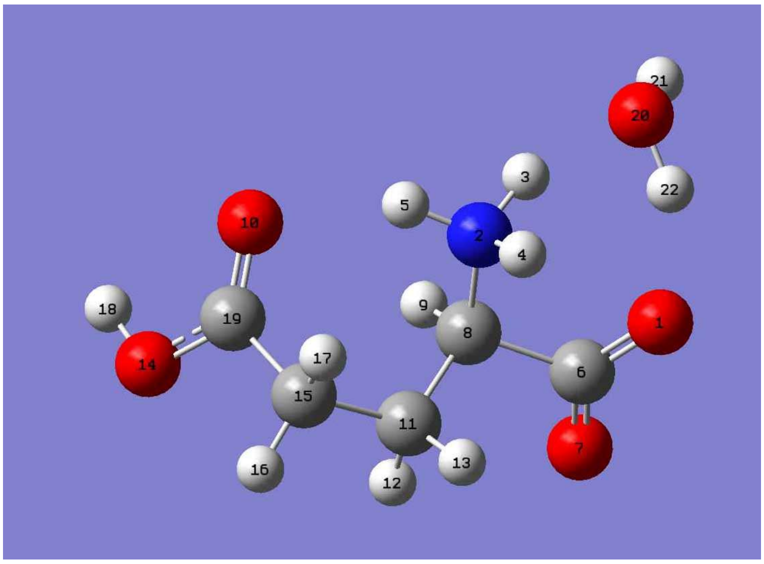

1. $S_1 = \Delta r(O14\text{-}H18)$

2. $S_2 = \Delta r(N2\text{-}H3) + \Delta r(N2\text{-}H4) + \Delta r(N2\text{-}H5)$

3. $S_3 = 2\ \Delta r(N2\text{-}H3) - \Delta r(N2\text{-}H4) - \Delta r(N2\text{-}H5)$

4. $S_4 = \Delta r(N2\text{-}H4) - \Delta r(N2\text{-}H5)$

5. $S_5 = \Delta r(C8\text{-}H9)$

6. $S_6 = \Delta r(C11\text{-}H12) + \Delta r(C11\text{-}H13)$

7. $S_7 = \Delta r(C11\text{-}H12) - \Delta r(C11\text{-}H13)$

8. $S_8 = \Delta r(C15\text{-}H16) + \Delta r(C15\text{-}H17)$

9. $S_9 = \Delta r(C15\text{-}H16) - \Delta r(C15\text{-}H17)$

10. $S_{10} = \Delta r(C19{=}O10)$

11. $S_{11} = \Delta r(C6\text{-}O1) - \Delta r(C6\text{-}O7)$

12. $S_{12} = \Delta r(C6\text{-}O1) + \Delta r(C6\text{-}O7)$

13. $S_{13} = \Delta r(C19\text{-}O14)$

14. $S_{14} = \Delta r(C6\text{-}C8)$

15. $S_{15} = \Delta r(C8\text{-}C11)$

16 $S_{16} = \Delta r(C11\text{-}C15)$

17. $S_{17} = \Delta r(C15\text{-}C19)$

18. $S_{18} = \Delta r(C8\text{-}N2)$

19. $S_{19} = \Delta\theta(C19\text{-}O18\text{-}H14)$

20. S_{20} = Δθ(O7=C6=O1)

21. S_{21} = Δθ(O10=C19-O14)

22. S_{22} = Δθ(O7=C6-C8)

23. S_{23} = Δθ(O10=C19-C15)

24. S_{24} = Δθ(N2-C8-C6)

25. S_{25} = Δθ(C6-C8-C11)

26. S_{26} = Δθ(C11-C15-C19)

27. S_{27} = Δθ(H3-N2-C8) + Δθ(H4-N2-C8) + Δθ(H5-N2-C8) - Δθ(H3-N2-H4) - Δθ(H4-N2-H5) - Δθ(H5-N2-H3)

28. S_{28} = 2 Δθ(H3-N2-C8) - Δθ(H4-N2-C8) - Δθ(H5-N2-C8)

29. S_{29} = Δθ(H4-N2-C8) - Δθ(H5-N2-C8)

30. S_{30} = Δθ(N2-C8-C11)

31. S_{31} = Δθ(H9-C8-C11)

32. S_{32} = Δθ(H9-C8-C6)

33. S_{33} = Δθ(H12-C11-C8) + Δθ(H13-C11-C8)

34. S_{34} = Δθ(H12-C11-C8) - Δθ(H13-C11-C8)

35. S_{35} = Δθ(H12-C11-C15) + Δθ(H13-C11-C15)

36. S_{36} = Δθ(H12-C11-C15) - Δθ(H13-C11-C15)

37. S_{37} = Δθ(H16-C15-C11) + Δθ(H17-C15-C11)

38. S_{38} = Δθ(H16-C15-C11) - Δθ(H17-C15-C11)

39. S_{39} = Δθ(H16-C15-C19) + Δθ(H17-C15-C19)

40. S_{40} = Δθ(H16-C15-C19) - Δθ(H17-C15-C19)

41. S_{41} = Δθ(C8-C11-C15)

42. S_{42} = 2 Δθ(H3-N2-H4) - Δθ(H4-N2-H5) -Δθ(H5-N2-H3)

43. S_{43} = Δθ(H4-N2-H5) -Δθ(H5-N2-H3)

44. S_{44} = Δτ(O1-C6-C8-C11)+ Δτ(O7=C6-C8-C11)+ Δτ(O1-C6-C8-N2)+ Δτ(N2-C8-C6=O7) + Δτ(H9-C8-C6=O7)

45. S_{45} = Δτ(N2-C8-C11-C15) + Δτ(H9-C8-C11-C15) +Δτ(C15-C11-C8-C6) + Δτ(H12-C11-C8-C6) + Δτ(H13-C11-C8-C6)

46. S_{46} = Δτ(H3-N2-C8-C11) + Δτ(H4-N2-C8-C11) + Δτ(H5-N2-C8-C11)

47. S_{47} = Δτ(C8-C11-C15-C19)

48. S_{48} = Δτ(H16-C15-C19=O10) + Δτ(H17-C15-C19=O10)

49. S_{49} = Δτ(H18-O14-C19=O10)

50. S_{50} = Δλ(O7=C6) out of plane

51. S_{51} = Δλ(O10=C19) out of plane

52. S_{52} = Δr(O20-H21)

53. S_{53} = Δr(O20-H22)

54. S_{54} = Δθ(H21-O20-H22)

55. S_{55} = Δr(H3···O20)

56. S_{56} = Δr(H22···O1)

57. S_{57} = Δτ(H22···O1=C6-C8)

58. S_{58} = Δθ(O20-H22···O1)

59. S_{59} = Δλ(H21-O20) out of plane

60. S_{60} = Δλ(H22-O20) out of plane

Supplementary Table S3. Potential energy distribution among normal modes of glutamic acid bound to a gold atom, calculated using BALGA program(G. Keresztury, G. Jalsovszky, J. Mol. Struct. 10 (1971) 304-305.; H. Rostkowski, L. Lapinski, M. Nowak, Vib. Spectrosc. 49 (2009) 43-51). List of internal coordinates is given below the table. Optimization of geometry and calculation of normal modes was performed using Gaussian with B3LYP/lanl2dz.

Mode number	Calculated (scaled by 0.968)	PED (%)	assignment
1.	3518	100 S_2	O14-H18 stretch.
2.	3461	92 S_4	asym. NH_2 stretch.
3.	3323	92 S_3	sym. NH_2 stretch.
4.	3167	100 S_1	O1-H5 stretch.
5.	3065	83 S_9	CH_2 asym. stretch.
6.	3045	88 S_7	CH_2 asym. stretch.
7.	2989	77 S_6 + 13 S_8	CH2 sym. stretch.
8.	2985	66 S_8 + 19 S_5	CH2 sym. stretch.
9.	2980	73 S_5 + 15 S_6	C8-H9 stretch
10.	1663	82 S_{10}	C6=O7 stretch.
11.	1640	44 S_{11} + 32 S_{42}	C19=O10 stretch. + H-N-H scissor.
12.	1605	45 S_{42} + 32 S_{11}	H-N-H scissor. + C19=O10 stretch.
13.	1465	34 S_{33} + 31 S_{39} + 19 S_{35}	CH_2 scissor.
14.	1461	32 S_{39} + 26 S_{33} + 19 S_{37} + 15 S_{35}	CH_2 scissor.
15.	1379	23 S_{35} + 16 S_{33} + 16 S_{37}	CH_2 wagg.
16.	1365	37 S_{32} + 25 S_{29}	H9-C8-C6 bend.
17.	1319	37 S_{31} + 15 S_{35} +10 S_{33}	HCC bend.
18.	1313	23 S_{47} + 17 S_{46} + 12 S_{44} + 11 S_{49}	CH_2 wagg.
19.	1293	27 S_{31} + 11 S_{35} + 10 S_{34}	HCC bend.
20.	1219	18 S_{34} + 15 S_{36} + 10 S_{12}	HCC bend.
21.	1198	25 S_{38} + 24 S_{40} + 14 S_{20}	CH_2 wagg + ..
22.	1190	38 S_{32} + 22 S_{29} + 11 S_{12} + 10 S_{19}	HCC + HNC bend.
23.	1163	47 S_{19} + 21 S_{29}	COH bend + HNC bend.
24.	1137	29 S_{19} + 25 S_{12} + ...	COH bend + C-C stretch
25.	1109	21 S_{18} + 16 S_{15} + 13 S_{20}	C-C stretch + COH bend.
26.	1079	28 S_{15} + 24 S_{16}	C-C stretch.
27.	1064	21 S_{13} + 18 S_{20} + 14 S_{18}	C-O stretch. + COH bend.
28.	999	25 S_{16} + 22 S_{28} + 10 S_{17}	C-C stretch + HNC bend.
29.	959	20 S_{34} + 13 S_{13} + 10 S_{40}	HCC bend.
30.	916	26 S_{18} + 15 S_{28} + 12 S_{38}	N-C stretch + HNC bend.

Table S3 continued

Mode number	Calculated (scaled by 0.968)	PED (%)	assignment
31.	874	22 S_{14} + 15 S_{12} + 14 S_{16} +13 S_{15}	C-C str.
32.	804	27 S_{17} + 14 S_{13} + ...	C-C + C-O stretch.
33.	745	15 S_{36}+ 12 S_{34} + 11 S_{38} + 10 S_{15}	CH_2 rock.
34.	714	34 S_{51} + 28 S_{49}	C19=O10 out of pl.
35.	691	26 S_{21} + 17 S_{50} + 15 S_{43}	O7=C6-O1 bend
36.	635	31 S_{43} + 23 S_{50}	O1-H5 torsion + O7-C6-C8-C11 torsion
37.	632	60 S_{49}	H18-O14 torsion
38.	611	42 S_{43} + 27 S_{46}	O1-H5 torsion
39.	555	28 S_{21} + 10 S_{50} + ...	O7=C6-O1 bend +C6=O7 out of pl.
40.	532	59 S_{22} + 10 S_{17}	O19=C10-O14 bend.
41.	476	29 S_{23} +19 S_{30} +...	O7=C6-C8 bend.
42.	429	30 S_{24} + 12 S_{21}	O10=C19-C15 bend.
43.	407	24 S_{30} + 19 S_{23} + 16 S_{24}	N2-C8-C6 bend.
44.	331	20 S_{34} + 12 S_{36} + 12 S_{41}	skeletal mode
45.	297	47 S_{35} + 12 S_{50}	CC str. + C6=O7 out of pl.
46.	209	53 S_{26} + 22 S_{23}	skeletal mode
47.	190	45 S_{17} +18 S_{45} + 17 S_{41}	C8-C11 torsion
48.	144	74 S_{47}	C8-C11-C15-C19 torsion
49.	110	21 S_{45} + 20 S_{52} + 19 S_{30} + 15 S_{53}	Au-N2 str.
50.	87	46 S_{52} + 34 S_{53} + 20 S_{54}	Au-N2-C8 bend.
51.	73	29 S_{45} + 18 S_{48} + 17 S_{54} + 13 S_{41}	torsion C8-C11 + torsion N2-C8
52.	44	54 S_{48} + 28 S_{45}	torsion around C15-C19
53.	35	90 S_{44}	torsion around C6-C8
54.	30	35 S_{54} + 14 S_{47} + 12 S_{52}	Au-N2-C8-C11 torsion

Definition of internal coordinates Glu-Au

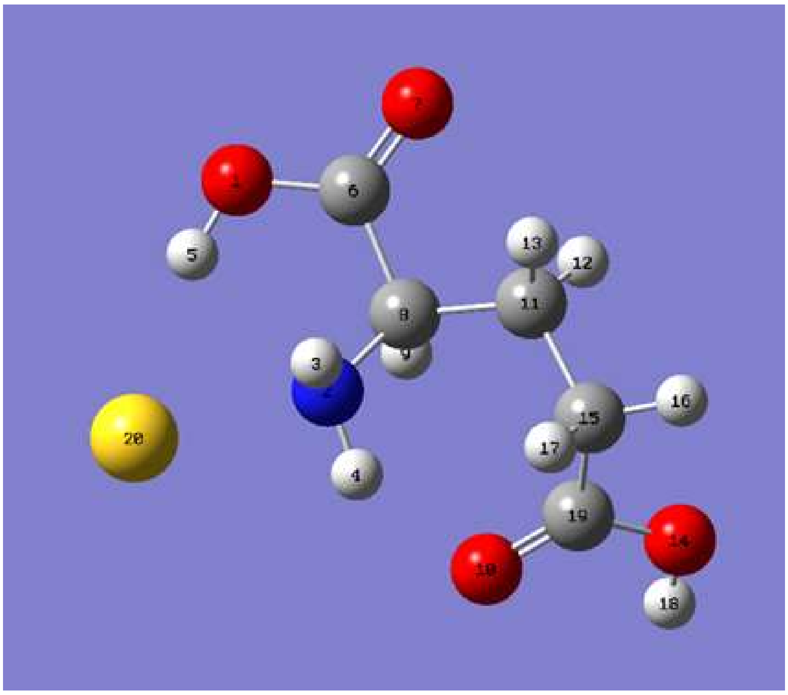

1. $S_1 = \Delta r(O1\text{-}H5)$

2. $S_2 = \Delta r(O14\text{-}H18)$

3. $S_3 = \Delta r(N2\text{-}H4) + \Delta r(N2\text{-}H3)$

4. $S_4 = \Delta r(N2\text{-}H4) - \Delta r(N2\text{-}H3)$

5. $S_5 = \Delta r(C8\text{-}H9)$

6. $S_6 = \Delta r(C11\text{-}H12) + \Delta r(C11\text{-}H13)$

7. $S_7 = \Delta r(C11\text{-}H12) - \Delta r(C11\text{-}H13)$

8. $S_8 = \Delta r(C15\text{-}H16) + \Delta r(C15\text{-}H17)$

9. $S_9 = \Delta r(C15\text{-}H16) - \Delta r(C15\text{-}H17)$

10. $S_{10} = \Delta r(C6{=}O7)$

11. $S_{11} = \Delta r(C19{=}O10)$

12. $S_{12} = \Delta r(C6\text{-}O1)$

13. $S_{13} = \Delta r(C19\text{-}O14)$

14. $S_{14} = \Delta r(C6\text{-}C8)$

15. $S_{15} = \Delta r(C8\text{-}C11)$

16 $S_{16} = \Delta r(C11\text{-}C15)$

17. $S_{17} = \Delta r(C15\text{-}C19)$

18. $S_{18} = \Delta r(C8\text{-}N2)$

19. $S_{19} = \Delta\theta$(C6-O1-H5)

20. $S_{20} = \Delta\theta$(C19-O14-H18)

21. $S_{21} = \Delta\theta$(O7=C6-O1)

22. $S_{22} = \Delta\theta$(O10=C19-O14)

23. $S_{23} = \Delta\theta$(O7=C6-C8)

24. $S_{24} = \Delta\theta$(O10=C19-C15)

25. $S_{25} = \Delta\theta$(N2-C8-C6)

26. $S_{26} = \Delta\theta$(C6-C8-C11)

27. $S_{27} = \Delta\theta$(C11-C15-C19)

28. $S_{28} = \Delta\theta$(H4-N2-C8) + $\Delta\theta$(H3-N2-C8)

29. $S_{29} = \Delta\theta$(H4-N2-C8) - $\Delta\theta$(H3-N2-C8)

30. $S_{30} = \Delta\theta$(N2-C8-C11)

31. $S_{31} = \Delta\theta$(H9-C8-C11)

32. $S_{32} = \Delta\theta$(H9-C8-C6)

33. $S_{33} = \Delta\theta$(H12-C11-C8) + $\Delta\theta$(H13-C11-C8)

34. $S_{34} = \Delta\theta$(H12-C11-C8) - $\Delta\theta$(H13-C11-C8)

35. $S_{35} = \Delta\theta$(H12-C11-C15) + $\Delta\theta$(H13-C11-C15)

36. $S_{36} = \Delta\theta$(H12-C11-C15) - $\Delta\theta$(H13-C11-C15)

37. $S_{37} = \Delta\theta$(H16-C15-C11) + $\Delta\theta$(H17-C15-C11)

38. $S_{38} = \Delta\theta$(H16-C15-C11) - $\Delta\theta$(H17-C15-C11)

39. $S_{39} = \Delta\theta$(H16-C15-C19) + $\Delta\theta$(H17-C15-C19)

40. $S_{40} = \Delta\theta$(H16-C15-C19) - $\Delta\theta$(H17-C15-C19)

41. $S_{41} = \Delta\theta$(C8-C11-C15)

42. $S_{42} = \Delta\theta$(H4-N2-H3)

43. $S_{43} = \Delta\tau$(H5-O1-C6=O7)

44. $S_{44} = \Delta\tau$(O1-C6-C8-C11)+ $\Delta\tau$(O7=C6-C8-C11)+ $\Delta\tau$(O1-C6-C8-N2

45. $S_{45} = \Delta\tau$(N2-C8-C11-C15) + $\Delta\tau$(H9-C8-C11-C15)

46. $S_{46} = \Delta\tau$(H4-N2-C8-C11) + $\Delta\tau$(H3-N2-C8-C11) + $\Delta\tau$(H4-N2-C8-C6) + $\Delta\tau$(H3-N2-C8-C6)

47. $S_{47} = \Delta\tau$(C8-C11-C15-C19) + $\Delta\tau$(H13-C11-C15-C19) + $\Delta\tau$(H12-C11-C15-C19) + $\Delta\tau$(H16-C15-C11-C8) + $\Delta\tau$(H17-C15-C11-C8)

48. S_{48} = Δτ(H16-C15-C19=O10) + Δτ(H17-C15-C19=O10)

49. S_{49} = Δτ(H18-O14-C19=O10)

50. S_{50} = Δλ(O7=C6) out of plane

51. S_{51} = Δλ(O10=C19) out of plane

52. S_{52} = Δθ(C8···H3···Au)

53. S_{53} = Δr(Au-N2)

54. S_{54} = Δτ(Au-N2-C8-C11)

Supplementary Table S4. Potential energy distribution among normal modes of glutamic acid bound to a silver atom, calculated using BALGA program(G. Keresztury, G. Jalsovszky, J. Mol. Struct. 10 (1971) 304-305.; H. Rostkowski, L. Lapinski, M. Nowak, Vib. Spectrosc. 49 (2009) 43-51). List of internal coordinates is given below the table. Optimization of geomtery and calculation of normal modes was performed using Gaussian with B3LYP/lanl2dz.

Mode number	Calculated (scaled by 0.968)	PED (%)	assignment
1.	3520	98 S_2	O14-H18 stretch.
2.	3518	97 S_4	asym. NH_2 stretch.
3.	3404	99 S_3	sym. NH_2 stretch.
4.	3064	86 S_9	CH2 asym. stretch.
5.	3040	73 S_1 + 19 S_7	O1-H3 stretch.
6.	3036	64 S_7 + 24 S_1	CH_2 asym. stretch.
7.	3000	94 S_5	C8-H9 stretch
8.	2985	83 S_8 + 12 S_9	CH2 sym. stretch.
9.	2968	84 S_6	CH2 sym. stretch.
10.	1694	80 S_{10}	C6=O7 stretch.
11.	1644	62 S_{42} + 21 S_{28}	H-N-H scissor.+ HNC bend.
12.	1634	65 S_{11} + 13 S_{42}	C19=O10 stretch.+ H-N-H scissor.
13.	1468	52 S_{33} + 41 S_{35}	CH_2 scissor.
14.	1457	61 S_{39} + 31 S_{37}	CH_2 scissor.
15.	1376	20 S_{37} + 19 S_{33} + 15 S_{35}	CH_2 wagg.
16.	1357	40 S_{31} + 27 S_{32} + 15 S_{29} + 10 S_{26}	H9-C8-C11 bend.
17.	1324	34 S_{19} + 11 S_{37} +11 S_{32}	H3-O1-C6 bend.
18.	1318	26 S_{19} + 17 S_{35} + 12 S_{37} + 10 S_{33}	CH_2 wagg.+ H3-O1-C6 bend.
19.	1305	13 S_{20} +11 S_{40} + 10 S_{13} + 10 S_{36}	H18-O14-C19 bend.+..
20.	1282	17 S_{32} + 17 S_{31} + 13 S_{34}	HCC bend.
21.	1219	28 S_{32} + 17 S_{34}+ 13 S_{29}	CH_2 twist + ..
22.	1201	26 S_{38} + 23 S_{40} + 17 S_{20}	CH_2 twist
23.	1146	24 S_{29} + 12 S_{31} + 11 S_{12}	HNC bend.
24.	1131	17 S_{20} + 17 S_{18} + 15 S_{36} + 10 S_{40}	H18-O14-C19 bend + C-C stretch
25.	1106	23 S_{12} + 22 S_{15} + 18 S_{23} + 16 S_{14}	C6-O1 stretch + COH bend.
26.	1061	27 S_{13} + 22 S_{20}	C19-O14 stretch.
27.	1044	27 S_{16} + + 13 S_{12} + 11 S_{29}	C-O stretch. + COH bend.
28.	970	22 S_{16} + 11 S_{17} + ...	C11-C15 + C15-C19 stretch.
29.	960	83 S_{43}	H3-O1-C6=O7 torsion
30.	948	15 S_{44} + 15 S_{40} + 14 S_{18} + 13 S_{38}	CH_2 rock. + N2-C8 stretch.

Table S4 cont.

Mode number	Calculated (scaled by 0.968)	PED (%)	assignment
31.	907	14 S_{16} + 11 S_{12} + 9 S_{14}	C11-C15 str.
32.	814	42 S_{28} + 14 S_{42} + 10 S_{14}	H-N-C bend.
33.	802	26 S_{17} + 11 S_{13} +..	C15-C19 str. + C19-O14 str.
34.	752	16 S_{36}+ 14 S_{38} + 10 S_{34}	CH2 rocking
35.	712	34 S_{51} + 27 S_{49}	O10=C19 outof pl.
36.	676	33 S_{50} + 18 S_{21}	O7=C6 out of pl.
37.	623	65 S_{49}	H18-O14 torsion
38.	561	36 S_{21} + 16 S_{50} + 10 S_{41}	O7=C6-O1 bend +C6=O7 out of pl.
39.	524	58 S_{22} + 10 S_{17}	O10=C19-O14 bend.
40.	507	43 S_{23} + 20 S_{25}	O7=C6-C8 bend.
41.	425	33 S_{24} +11 S_{22}	O10=C19-C15 bend
42.	403	47 S_{46} + 25 S_{30}	torsion around N2-C8
43.	389	32 S_{46} + 29 S_{30} + 12 S_{24}	N2-C8-C11 bend + torsion around N2-C8
44.	342	28 S_{25} + 20 S_{21}	N-C8-C6 bend
45.	302	20 S_{24} + 16 S_{25} +13 S_{27} + 10 S_{23}	O10=C19-C15 bend+ NCC bend.
46.	201	33 S_{27} + 29 S_{26}	C11-C15-C19 bend.
47.	161	34 S_{41} +19 S_{26} +11 S_{47} + 11 S_{27}	C8-C15-C11 bend.
48.	113	46 S_{47} + 18 S_{44}	C8-C11-C15-C19 torsion
49.	86	58 S_{44} + 24 S_{45}	torsion around C6-C8
50.	56	67 S_{53} + 16 S_{45}	Ag ···O1 stretch.
51.	42	27 S_{45} + 17 S_{47} + 16 S_{52} + 12 S_{44}	torsion around C8-C11+ torsion C11-C15
52.	33	68 S_{48}	torsion around C15-C19
53.	19	78 S_{52}	Ag ···O1-C6 bend
54.	8	97 S_{54}	Ag ··· O1-C6-C8 torsion

Definition of internal coordinates Glu-Ag

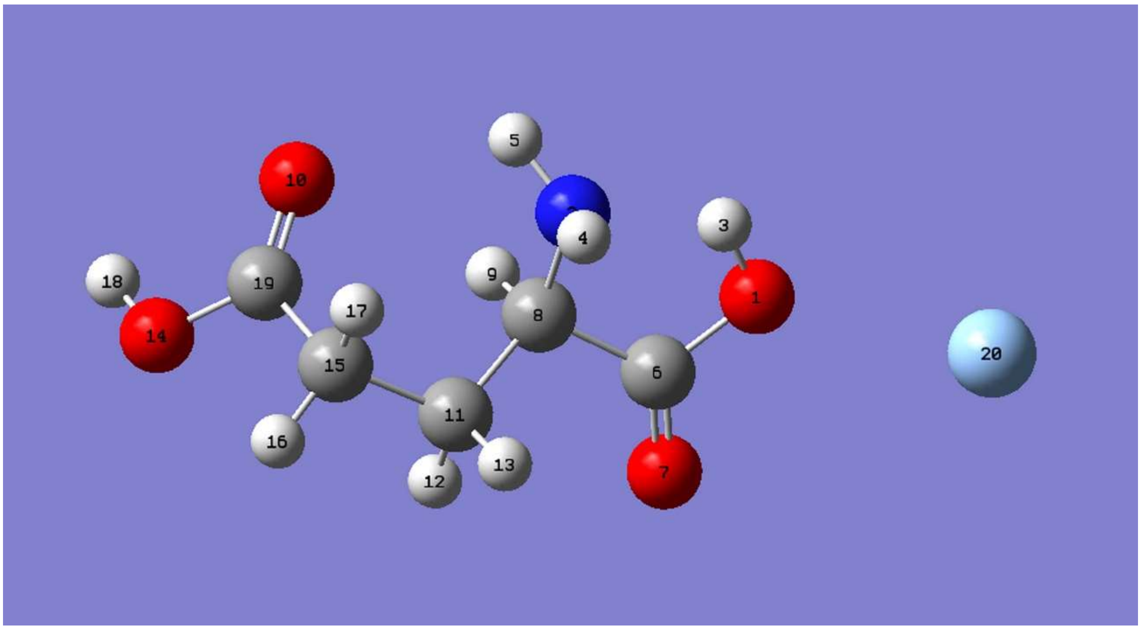

1. $S_1 = \Delta r(O1\text{-}H3)$

2. $S_2 = \Delta r(O14\text{-}H18)$

3. $S_3 = \Delta r(N2\text{-}H4) + \Delta r(N2\text{-}H5)$

4. $S_4 = \Delta r(N2\text{-}H4) - \Delta r(N2\text{-}H5)$

5. $S_5 = \Delta r(C8\text{-}H9)$

6. $S_6 = \Delta r(C11\text{-}H12) + \Delta r(C11\text{-}H13)$

7. $S_7 = \Delta r(C11\text{-}H12) - \Delta r(C11\text{-}H13)$

8. $S_8 = \Delta r(C15\text{-}H16) + \Delta r(C15\text{-}H17)$

9. $S_9 = \Delta r(C15\text{-}H16) - \Delta r(C15\text{-}H17)$

10. $S_{10} = \Delta r(C6{=}O7)$

11. $S_{11} = \Delta r(C19{=}O10)$

12. $S_{12} = \Delta r(C6\text{-}O1)$

13. $S_{13} = \Delta r(C19\text{-}O14)$

14. $S_{14} = \Delta r(C6\text{-}C8)$

15. $S_{15} = \Delta r(C8\text{-}C11)$

16 $S_{16} = \Delta r(C11\text{-}C15)$

17. $S_{17} = \Delta r(C15\text{-}C19)$

18. $S_{18} = \Delta r(C8\text{-}N2)$

19. $S_{19} = \Delta\theta(C6\text{-}O1\text{-}H3)$

20. $S_{20} = \Delta\theta(C19\text{-}O14\text{-}H18)$

21. $S_{21} = \Delta\theta(O7{=}C6\text{-}O1)$

22. $S_{22} = \Delta\theta(O10{=}C19\text{-}O14)$

23. $S_{23} = \Delta\theta(O7{=}C6\text{-}C8)$

24. $S_{24} = \Delta\theta(O10{=}C19\text{-}C15)$

25. $S_{25} = \Delta\theta(N2\text{-}C8\text{-}C6)$

26. $S_{26} = \Delta\theta(C6\text{-}C8\text{-}C11)$

27. $S_{27} = \Delta\theta(C11\text{-}C15\text{-}C19)$

28. $S_{28} = \Delta\theta(H4\text{-}N2\text{-}C8) + \Delta\theta(H5\text{-}N2\text{-}C8)$

29. $S_{29} = \Delta\theta(H4\text{-}N2\text{-}C8) - \Delta\theta(H5\text{-}N2\text{-}C8)$

30. $S_{30} = \Delta\theta(N2\text{-}C8\text{-}C11)$

31. $S_{31} = \Delta\theta(H9\text{-}C8\text{-}C11)$

32. $S_{32} = \Delta\theta(H9\text{-}C8\text{-}C6)$

33. $S_{33} = \Delta\theta(H12\text{-}C11\text{-}C8) + \Delta\theta(H13\text{-}C11\text{-}C8)$

34. $S_{34} = \Delta\theta(H12\text{-}C11\text{-}C8) - \Delta\theta(H13\text{-}C11\text{-}C8)$

35. $S_{35} = \Delta\theta(H12\text{-}C11\text{-}C15) + \Delta\theta(H13\text{-}C11\text{-}C15)$

36. $S_{36} = \Delta\theta(H12\text{-}C11\text{-}C15) - \Delta\theta(H13\text{-}C11\text{-}C15)$

37. $S_{37} = \Delta\theta(H16\text{-}C15\text{-}C11) + \Delta\theta(H17\text{-}C15\text{-}C11)$

38. $S_{38} = \Delta\theta(H16\text{-}C15\text{-}C11) - \Delta\theta(H17\text{-}C15\text{-}C11)$

39. $S_{39} = \Delta\theta(H16\text{-}C15\text{-}C19) + \Delta\theta(H17\text{-}C15\text{-}C19)$

40. $S_{40} = \Delta\theta(H16\text{-}C15\text{-}C19) - \Delta\theta(H17\text{-}C15\text{-}C19)$

41. $S_{41} = \Delta\theta(C8\text{-}C11\text{-}C15)$

42. $S_{42} = \Delta\theta(H4\text{-}N2\text{-}H5)$

43. $S_{43} = \Delta\tau(H3\text{-}O1\text{-}C6{=}O7)$

44. $S_{44} = \Delta\tau(O1\text{-}C6\text{-}C8\text{-}C11) + \Delta\tau(O7{=}C6\text{-}C8\text{-}C11) + \Delta\tau(O1\text{-}C6\text{-}C8\text{-}N2)$

45. $S_{45} = \Delta\tau(N2\text{-}C8\text{-}C11\text{-}C15) + \Delta\tau(H9\text{-}C8\text{-}C11\text{-}C15)$

46. $S_{46} = \Delta\tau(H4\text{-}N2\text{-}C8\text{-}C11) + \Delta\tau(H5\text{-}N2\text{-}C8\text{-}C11) + \Delta\tau(H4\text{-}N2\text{-}C8\text{-}C6) + \Delta\tau(H5\text{-}N2\text{-}C8\text{-}C6)$

47. S_{47} = Δτ(C8-C11-C15-C19) + Δτ(H13-C11-C15-C19) + Δτ(H12-C11-C15-C19) + Δτ(H16-C15-C11-C8) + Δτ(H17-C15-C11-C8)

48. S_{48} = Δτ(H16-C15-C19=O10) + Δτ(H17-C15-C19=O10)

49. S_{49} = Δτ(H18-O14-C19=O10)

50. S_{50} = Δλ(O7=C6) out of plane

51. S_{51} = Δλ(O10=C19) out of plane

52. S_{52} = Δθ(C6-O1···Ag)

53. S_{53} = Δr(Ag···O1)

54. S_{54} = Δτ(Ag···O1-C6-C8)

Supplementary Table S5. Potential energy distribution among normal modes of sodium glutamate calculated using BALGA program(G. Keresztury, G. Jalsovszky, J. Mol. Struct. 10 (1971) 304-305.; H. Rostkowski, L. Lapinski, M. Nowak, Vib. Spectrosc. 49 (2009) 43-51). List of internal coordinates is given below the table. Optimization of geomtery and calculation of normal modes was performed using Gaussian with B3LYP/6-311++G(d,p).

Mode number	Calculated (scaled by 0.968)	PED (%)	assignment
1.	3635	100 S_2	O14-H18 stretch.
2.	3460	96 S_4	asym. NH_2 stretch.
3.	3379	97 S_3	sym. NH_2 stretch.
4.	3012	70 S_7 + 15 S_6 + 10 S_5	CH_2 asym. stretch.
5.	2998	79 S_5	C8-H9 stretch
6.	2991	75 S_9	CH_2 asym. stretch.
7.	2954	87 S_8	CH_2 sym. stretch.
8.	2937	72 S_6 + 18 S_7	CH_2 stretch.
9.	1753	84 S_{11}	C19=O10 stretch
10.	1626	76 S_{42} + 23 S_{28}	H-N-H scissor.+ H-N-C bend.
11.	1539	52 S_{10} + 36 S_{12}	antisym. CO_2^- stretch
12.	1437	58 S_{35} + 36 S_{33}	CH_2 scissoring
13.	1408	71 S_{39} + 20 S_{37}	CH_2 scissoring
14.	1392	32 S_{12} + 12 S_{34} + 13 S_{10}	CO_2^- sym. stretch.
15.	1363	50 S_{37} + 11 S_{17}	HCC bend.
16.	1347	26 S_{31} + 18 S_{29} +18 S_{32}	H9-C8-C11 bend.
17.	1334	46 S_{33}	CH_2 twist
18.	1286	30 S_{34} + 15 S_{31} + 10 S_{29}	CH_2 wagg.
19.	1256	44 S_{20} + 11 S_{22}	C-O14-H18 bend.
20.	1218	40 S_{32} + 14 S_{29}	H9-C8-C6 bend.
21.	1202	33 S_{40} + 20 S_{38}	CH_2 twist
22.	1144	21 S_{36} + 17 S_{29} + 10 S_{40}	CH_2 twist + HNC bend.
23.	1107	34 S_{18} + 16 S_{15} + ..	N2-C8 stretch.
24.	1091	34 S_{13} + 16 S_{20} + ..	C19-O14 stretch.
25.	1041	40 S_{16} + ..	C-C stretch
26.	963	20 S_{16} + 15 S_{28} + ...	C-C stretch. + HNC bend.
27.	952	13 S_{15} + 11 S_{38} + ..	C11-C15 stretch. +HCC bend.
28.	921	18 S_{34} + 16 S_{14} + 13 S_{18}	CH2 rock + C6-C8 stretch.
29.	864	36 S_{28} + 11 S_{18} + 10 S_{21}	NH_2 wagg.

Table S5 continued

Mode number	Calculated (scaled by 0.968)	PED (%)	assignment
30.	820	22 S_{17} + 15 S_{50}	C-C stretch
31.	786	21 S_{15} + 18 S_{21} + 16 S_{17}	C-C str. + OCC bend.
32.	741	24 S_{50} + 17 S_{21} +..	C6=O7 out of pl.
33.	680	25 S_{21} + 19 S_{50}	O=C=O- bend.
34.	631	69 S_{49}	OH torsion
35.	603	26 S_{24} + 20 S_{22} + 11 S_{49}	O10=C19-C15 bend.
36.	527	33 S_{23} + 12 S_{22}	N2-C8-C6 bend.
37.	501	33 S_{51} + 17 S_{22}	H18-O14-C19=O10 torsion
38.	473	30 S_{51} +...	O10=C19 out of pl.
39.	403	39 S_{30} + 15 S_{1}	N2-C8-C11 bend.
40.	356	34 S_{1} + 21 S_{23} + 14 S_{30}	Na···O1 stretch.
41.	308	48 S_{19} + 22 S_{25}	Na···O1=C6 bend.
42.	266	32 S_{24} + 22 S_{41} + 14 S_{27}	O10-C19-C15 bend.
43.	227	24 S_{41} + 17 S_{27} + 16 S_{45}	C8-C11-C15 bend.
44.	211	64 S_{46}	torsion around N2-C8
45.	199	28 S_{26} + 24 S_{46} + 14 S_{1} + 11 S_{43}	C11-C8-C6 bend.+..
46.	169	21 S_{19} + 19 S_{23} + 14 S_{26} + 10 S_{25}	skeletal mode
47.	86	59 S_{47} +10 S_{27}	torsion around C11-C15
48.	72	56 S_{43} + 15 S_{44} + 12 S_{50}	Na-O1-C6-O7 torsion
49.	40	66 S_{45} + 10 S_{44}	torsion around C8-C11
50.	35	40 S_{44} + 36 S_{48} + 13 S_{47}	torsion around C6-C8
51.	27	73 S_{48} + 27 S_{44}	torsion around C15-C19

Definition of internal coordinates of sodium glutamate

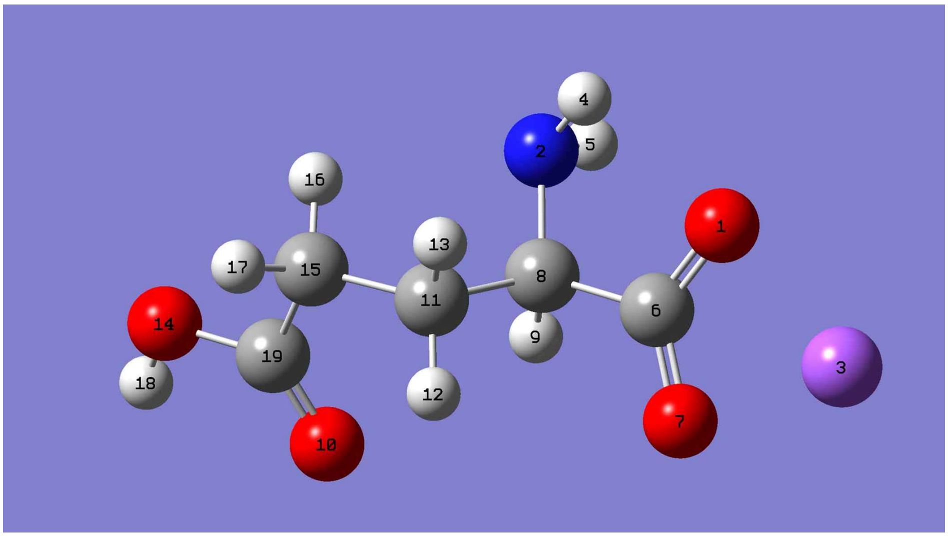

1. $S_1 = \Delta r(O1\text{-}Na)$

2. $S_2 = \Delta r(O14\text{-}H18)$

3. $S_3 = \Delta r(N2\text{-}H4) + \Delta r(N2\text{-}H5)$

4. $S_4 = \Delta r(N2\text{-}H4) - \Delta r(N2\text{-}H5)$

5. $S_5 = \Delta r(C8\text{-}H9)$

6. $S_6 = \Delta r(C11\text{-}H12) + \Delta r(C11\text{-}H13)$

7. $S_7 = \Delta r(C11\text{-}H12) - \Delta r(C11\text{-}H13)$

8. $S_8 = \Delta r(C15\text{-}H16) + \Delta r(C15\text{-}H17)$

9. $S_9 = \Delta r(C15\text{-}H16) - \Delta r(C15\text{-}H17)$

10. $S_{10} = \Delta r(C6{=}O7)$

11. $S_{11} = \Delta r(C19{=}O10)$

12. $S_{12} = \Delta r(C6\text{-}O1)$

13. $S_{13} = \Delta r(C19\text{-}O14)$

14. $S_{14} = \Delta r(C6\text{-}C8)$

15. $S_{15} = \Delta r(C8\text{-}C11)$

16 $S_{16} = \Delta r(C11\text{-}C15)$

17. $S_{17} = \Delta r(C15\text{-}C19)$

18. $S_{18} = \Delta r(C8\text{-}N2)$

19. S_{19} = Δθ(C6-O1-Na)

20. S_{20} = Δθ(C19-O14-H18)

21. S_{21} = Δθ(O7=C6-O1)

22. S_{22} = Δθ(O10=C19-O14)

23. S_{23} = Δθ(O7=C6-C8)

24. S_{24} = Δθ(O10=C19-C15)

25. S_{25} = Δθ(N2-C8-C6)

26. S_{26} = Δθ(C6-C8-C11)

27. S_{27} = Δθ(C11-C15-C19)

28. S_{28} = Δθ(H4-N2-C8) + Δθ(H5-N2-C8)

29. S_{29} = Δθ(H4-N2-C8) - Δθ(H5-N2-C8)

30. S_{30} = Δθ(N2-C8-C11)

31. S_{31} = Δθ(H9-C8-C11)

32. S_{32} = Δθ(H9-C8-C6)

33. S_{33} = Δθ(H12-C11-C8) + Δθ(H13-C11-C8)

34. S_{34} = Δθ(H12-C11-C8) - Δθ(H13-C11-C8)

35. S_{35} = Δθ(H12-C11-C15) + Δθ(H13-C11-C15)

36. S_{36} = Δθ(H12-C11-C15) - Δθ(H13-C11-C15)

37. S_{37} = Δθ(H16-C15-C11) + Δθ(H17-C15-C11)

38. S_{38} = Δθ(H16-C15-C11) - Δθ(H17-C15-C11)

39. S_{39} = Δθ(H16-C15-C19) + Δθ(H17-C15-C19)

40. S_{40} = Δθ(H16-C15-C19) - Δθ(H17-C15-C19)

41. S_{41} = Δθ(C8-C11-C15)

42. S_{42} = Δθ(H4-N2-H5)

43. S_{43} = Δτ(Na-O1-C6=O7)

44. S_{44} = Δτ(O1-C6-C8-C11)+ Δτ(O7=C6-C8-C11)+ Δτ(O1-C6-C8-N2)+ Δτ(N2-C8-C6=O7) + Δτ(H9-C8-C6=O7)

45. S_{45} = Δτ(N2-C8-C11-C15) + Δτ(H9-C8-C11-C15) + Δτ(C15-C11-C8-C6) + Δτ(H12-C11-C8-C6) + Δτ(H13-C11-C8-C6)

46. S_{46} = Δτ(H4-N2-C8-C11) + Δτ(H5-N2-C8-C11) + Δτ(H4-N2-C8-C6) + Δτ(H5-N2-C8-C6)

47. S_{47} = Δτ(C8-C11-C15-C19)

48. $S_{48} = \Delta\tau$(H16-C15-C19=O10) + $\Delta\tau$(H17-C15-C19=O10)

49. $S_{49} = \Delta\tau$(H18-O14-C19=O10)

50. $S_{50} = \Delta\lambda$(O7=C6) out of plane

51. $S_{51} = \Delta\lambda$(O10=C19) out of plane
